# Urine Metabolomics for Renal Cell Carcinoma (RCC) Prediction: Tryptophan Metabolism as an Important Pathway in RCC

**DOI:** 10.3389/fonc.2019.00663

**Published:** 2019-07-17

**Authors:** Xiaoyan Liu, Mingxin Zhang, Xiang Liu, Haidan Sun, Zhengguang Guo, Xiaoyue Tang, Zhan Wang, Jing Li, Hanzhong Li, Wei Sun, Yushi Zhang

**Affiliations:** ^1^School of Basic Medicine, Institute of Basic Medical Sciences, Peking Union Medical College, Chinese Academy of Medical Sciences, Beijing, China; ^2^Department of Urology, Peking Union Medical College Hospital, Chinese Academy of Medical Science, Beijing, China; ^3^Department of Urology, The Affiliated Hospital of Qingdao University, Qingdao, China

**Keywords:** renal cell carcinoma, metabolomics, benign tumors, biomarker, tryptophan metabolism

## Abstract

Renal cell carcinoma (RCC) is the second most lethal urinary cancer. RCC is frequently asymptomatic and it is already metastatic at diagnosis. There is an urgent necessity for RCC specific biomarkers selection for diagnostic and prognostic purposes. In present study, we applied liquid chromatography—mass spectrometry (LC-MS) based metabolomics to analyze urine samples of 100 RCC, 34 benign kidney tumors and 129 healthy controls. Differential metabolites were analyzed to investigate if urine metabolites could differentiate RCC from non-RCC. A panel consisting of 9 metabolites showed the best predictive ability for RCC from the health controls with an area under the curve (AUC) values of 0.905 for the training dataset and 0.885 for the validation dataset. Separation was observed between the RCC and benign samples with an AUC of 0.816. RCC clinical stages (T1 and T2 vs. T3 and T4) could be separated using a panel of urine metabolites with an AUC of 0.813. One metabolite, N-formylkynurenine, was discovered to have potential value for RCC diagnosis from non-RCC subjects with an AUC of 0.808. Pathway enrichment analysis indicated that tryptophan metabolism was an important pathway in RCC. Our data concluded that urine metabolomics could be used for RCC diagnosis and would provide candidates for further targeted metabolomics analysis of RCC.

## Introduction

Renal cell carcinoma (RCC) is the second most lethal urinary cancer and accounts for 5% of all adult malignancies (1). Clinically, dynamic contrast-enhanced computed tomography (CT) provides an accurate diagnosis of RCC in most cases. However, some small carcinomas are difficult to confirm. Furthermore, differentiating benign kidney tumors from RCC still remains a clinical challenge, even when images are re-examined by experienced radiologists. Final confirmation of RCC requires pathological examination of puncture or surgical resection. Approximately 20–30% of small renal masses that are surgically removed are found to be benign (2). Thus, the development of new, accurate, non-invasive diagnostic methods will have an important impact in RCC clinical management in its earliest stage and could reduce unnecessary treatment for benign tumors and increase the chance of nephron-sparing treatment.

RCC as a metabolic disease is well-suited to metabolomic analysis. Understanding and measuring metabolic status variations accompanying disease progression would be a useful strategy for potential new diagnostic biomarker discovery. Metabolomic analysis of urine, which is obviously closely related to RCC status, is an ideal non-invasive means to explore RCC (3). In 2011, Kim et al. performed a metabolomic analysis of urine from 29 RCC patients using LC-MS and GC-MS and found that quinolinate, 4-hydroxybenzoate, and gentisate are differentially expressed (4). In 2012, Ganti et al. utilized metabolomics to evaluate compounds appearing in the urine of kidney cancer patients (29 subjects) and control patients (33 subjects). Several acylcarnitines were discovered as a function of both cancer status and kidney cancer grade, with most of the acylcarnitine levels showing an increase in the urine of cancer patients (5). In 2016, Monteiro et al. analyzed the urine metabolome of RCC patients (*n* = 42) and controls (*n* = 49) using nuclear magnetic resonance (NMR) spectroscopy. A 32-metabolite/resonance signature, including 2-KG, N-methyl-2-pyridone-5-carboxamide (2-Py), bile acids, galactose, hypoxanthine, isoleucine, pyruvate, and succinate, could successfully distinguish RCC patients from controls (6).

The research described above applied urine metabolomics for RCC diagnosis from healthy controls based on a small sample size, and more samples are necessary to discover and validate RCC biomarkers. In addition, to our knowledge, there are still no studies that present urine metabolome differences between RCC and benign tumors. In the present work, we applied LC-MS-based metabolomics to analyse 263 urine samples from Chinese subjects, including 100 RCCs, 34 benign controls and 129 healthy controls, to investigate whether metabolic profiles could differentiate RCC from non-RCC samples (including healthy and benign controls). Furthermore, a study was also performed to distinguish RCC stages (T1 and T2 vs. T3 and T4) using urine metabolomics. This study will provide new insights into the diagnosis of RCC and possible clues for a metabolic mechanism.

## Materials and Methods

### Sample Collection

First morning urine (midstream) samples were collected from ~07:00 to 09:00 a.m. on an empty stomach from two cohorts: the training set includes 67 RCC patients, 34 benign tumor patients and 96 healthy human adults, while the test set includes 33 RCC patients, 7 benign tumor patients and 33 healthy controls. All cases were from Beijing Union Hospital. These groups did not include subjects suffering from any acute conditions or taking any medications in the latest 3 months. The glomerular filtration rate (GFR) and urine protein content of RCC patients were within normal ranges. The healthy control subjects were enrolled with matched genders and ages with the RCC patients to reduce interference from physiological factors. Both informed verbal and written consent were obtained from the subjects before participating in this study. RCC was diagnosed by a pathological investigation and graded according to the Union for International Cancer Control (UICC) tumor node-metastasis (TNM) staging system. RCC without metastases (T1–2, limited to the kidney) was categorized as early stage and RCC with metastases (T3–4) as late stage. The detailed demographics are shown in Table 1 and Table S1.

**Table 1:** Subjects information.

	**Cohort 1 (training set)**		
	**RCC (F/M)**	**Healthy control (F/M)**	**Benign (F/M)**
# of subjects	67 (19/48)	96 (35/61)	34 (20/14)
Age	53.5 ± 14.7	54.8 ± 11.5	46.4 ± 11.5
BMI	24.7 ± 3.7	22.7 ± 1.8	24.8 ± 3.6
eGFR (ml/min/1.7312)	98.2 ± 13.8	102.9 ± 10.5	98.1 ± 12.3
	**Cohort 2 (validation set)**		
# of subjects	33 (4/29)	33 (6/27)	7 (2/5)
Age	50.0 ± 14.3	51.5 ± 16.4	49.1 ± 8.7
BMI	25.6 ± 3.2	22.8 ± 2.4	24.5 ± 1.8
eGFR (ml/min/1.7312)	102.4 ± 10.3	105.6 ± 10.4	99.8 ± 11.7
	**RCC Staging (Combination of cohort 1 and 2)**		
Early (T1 and T2)	86 (18/68)		
Late (T3 and T4)	14 (5/9)		

### Sample Preparation

Urine sample preparation was performed based on previous methods (7). In brief, acetonitrile (200 μl) was added to each urine sample (200 μl), then the mixture was vortexed for 30 s and centrifuged at 14,000 × g for 10 min. The supernatant was dried under vacuum and then reconstituted with 200 μl of 2% acetonitrile. Urinary metabolites were further separated from larger molecules using 10 kDa molecular weight cut-off ultracentrifugation filters (Millipore Amicon Ultra, MA) before being transferred to the autosamplers. The quality control (QC) sample was a pooled urine sample prepared by mixing aliquots of fifty representative samples across different groups to be analyzed and was therefore globally representative of the whole sample set. The QC samples were injected every 10 samples throughout the analytical run to provide a set of data from which method stability and repeatability can be assessed.

### LC-MS Analysis

Ultra-performance LC-MS analyses of samples were conducted using a Waters ACQUITY H-class LC system coupled with an LTQ-Orbitrap Velos pro mass spectrometer (Thermo Fisher Scientific, MA. USA). Detailed parameters are listed in the Supplementary Methods.

### Statistical Data Analysis

Raw data files were processed by Progenesis QI (Version 2.0, Nonlinear Dynamics) software. The detailed processing parameters are provided in the Supplementary Methods file. The raw data file exported from QI was further processed by MetaboAnalyst 3.0 (http://www.metaboanalyst.ca), including missing value estimation, log2 transformation and Pareto scaling. Variables missed in 50% or greater of all samples were removed from further statistical analysis. Pattern recognition analysis (principal component analysis, PCA; orthogonal partial least squares discriminant analysis, OPLS-DA) was performed based on a training set using SIMCA 14.0 (Umetrics, Sweden) software. One hundred permutation validations were performed to evaluate the fitting of the OPLS-DA model. Variable importance for the projection (VIP) values obtained from the OPLS-DA were used for differential metabolite selection. Non-parametric tests (Wilcoxon rank-sum test) were used to evaluate the significance of the variables. False discovery rate (FDR) correction was used to estimate the chance of false positives and correct for multiple hypothesis testing. Differential metabolites were selected from the training set according to the criteria: (1) VIP value above 1; and (2) adjusted *p*-value below 0.05. These metabolites were further validated in the validation set. Features showing significant differences in both training and validation sets were considered as disease-related. An ROC curve was constructed based on differential metabolites selected from the training set using logistic regression algorithm. For RCC vs. healthy controls and RCC vs. non-RCCs, an external validation set was used to validate the accuracy of the potential biomarker panels. However, for RCC vs. benign and RCC early stage vs. late stage, 10-fold cross-validation was performed in the training set. These model constructions and validation were carried out based on MetaboAnalyst 3.0 platforms.

### Metabolite Annotation and Pathway Analysis

Metabolic pathways and predicted metabolites in the pathways were analyzed using the “Mummichog” algorithm based on the MetaboAnalyst 3.0 platform. The detailed parameters for the Mummichog analysis were provided in the Supplementary Methods. Metabolite annotation was further determined from the exact mass composition, from the goodness of isotopic fit for the predicted molecular formula and from MS/MS fragmentation comparing hits with databases (HMDB http://www.hmdb.ca/), thus qualifying for annotation at MSI level II using Progenesis QI. For endogenous metabolites lacking a chemical formula, the accurate molecular mass was given based on the calculated isotopic features and ion adducts. Detailed methods were listed in the Supplementary Methods.

## Results

**Figure 1:**
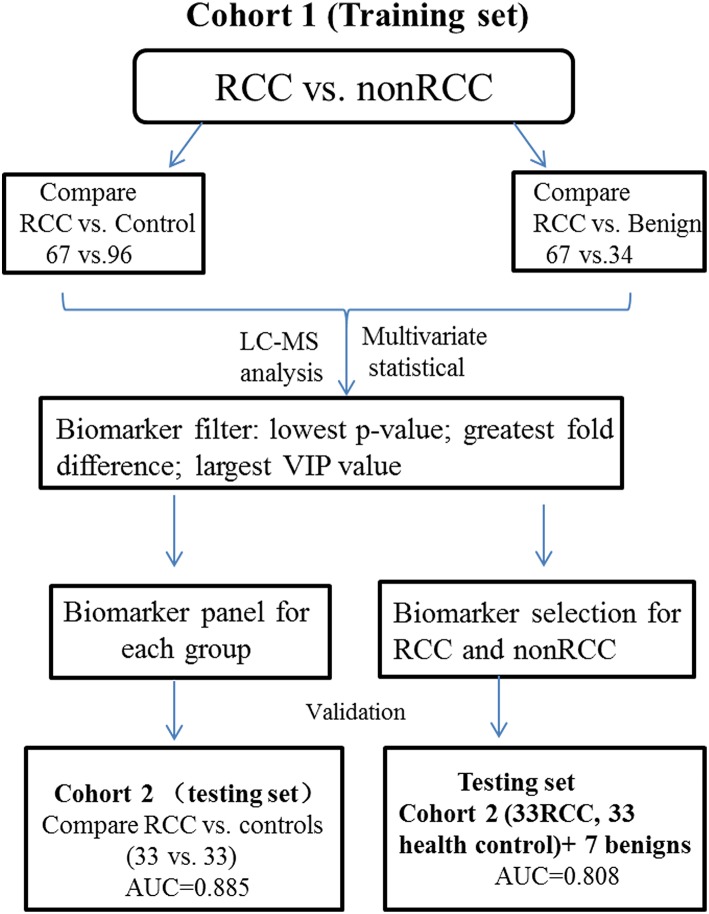
Study design for RCC distinction from control and benign subjects.

The major objective of our study was to discover potential biomarkers to distinguish RCC not only from healthy controls but also from subjects with benign tumors. The experimental strategy is shown in Figure 1. Metabolite variation and pathway regulation associated with RCC were explored based on analysis of metabolic profile differences between RCC and healthy and benign controls. Potential biomarkers for RCC were further explored based on differential metabolites and were validated using 10-fold cross validation or external validation.

### Quality Control

This cohort of samples was analyzed randomly, which took almost a week. Tight clustering of QC samples (Figure S1) demonstrated the quality of the QC data and essential repeatability and stability throughout the analytical run.

### Distinguishing RCC From Healthy Controls by Urine Metabolomics

**Figure 2:**
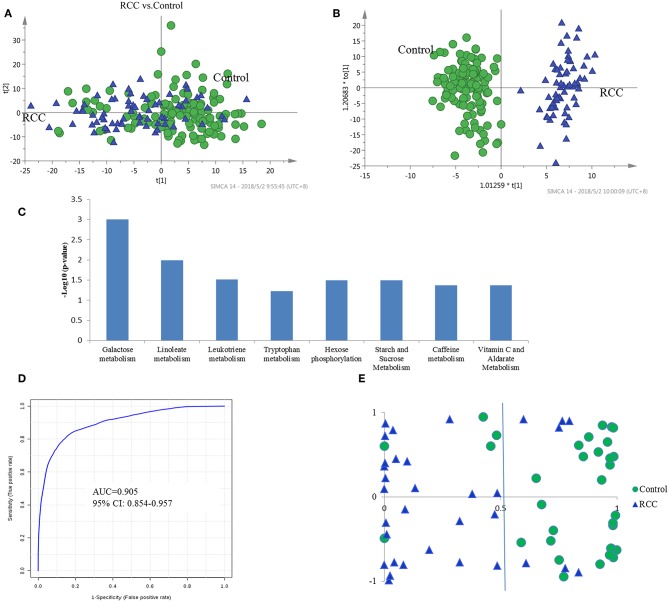
Analysis of urine metabolomic of RCC and health control. **(A)** Score plot of unsupervised PCA overview of urinary metabolic profiling of RCC and control in training set. **(B)** OPLS-DA model based on human urine for classification of RCC and control in training set. **(C)** Shifted metabolic pathways in RCC, when compared with the healthy controls. These pathways were enriched by using Mummichog algorithm. The smaller the *p*-value is, the higher confidence the pathway have. **(D)** ROC plot with 10-fold cross-validation in training set for distinction of RCC and control based on metabolite panel in Table S5. **(E)** Prediction accuracy of RCC prediction model established by a metabolite panel in Table S4 in validation cohort.

LC-MS-based urine samples from RCC and controls yielded 2,500 spectral features after QC filtering. Apparent differences between the metabolic profiles of RCC and the healthy controls were observed from the PCA score plot (Figure 2A). Furthermore, the OPLS-DA model achieved better separation (Figure 2B). Permutation tests were performed to confirm the stability and robustness of the supervised models presented in this study (Figure S2). In total, 455 differential metabolites were assigned, contributing to group separation, among which, 145 metabolites were also significantly changed in the validation set. These metabolites were further submitted for pathway analysis and prediction model construction.

Pathway enrichment analysis using the Mummichog algorithm showed significant enrichment (*p* < 0.05) of several pathways in the RCC group compared with those in healthy controls, including galactose metabolism, linoleate metabolism, leukotriene metabolism, tryptophan metabolism, etc. (Figure 2C; Table S2). The predicted activity network is shown in Figure S3. Mutual regulation of these disturbed pathways contributes to metabolic disorder in RCC.

Annotation of the top discriminatory features followed by MS/MS evaluation showed 65 metabolites were significantly differentially detected between RCC and healthy control samples (Table S3). Metabolites with higher levels in RCC included steroids such as androstenedione, alpha-CEHC, and 19-nor-5-androstenediol, dipeptides such as aspartyl-phenylalanine and glutamyl-threonine, bile acid metabolites such as 7-alpha-hydroxy-3-oxochol-4-en-24-oic acid and lithocholyltaurine, and exogenous sulfate metabolites. On the other hand, metabolites with lower levels included steroid glucuronidation metabolites such as tetrahydroaldosterone-3-glucuronide and cortolone-3-glucuronide, indicating a potential roles for glucuronidation in RCC development. Moreover, a caffeine metabolite (methylxanthine) was found to be decreased in RCC patients, which pinpoints aberrations in xenobiotic metabolism. A free fatty acid (11-dodecenoic acid) showed higher levels in RCC, and the oxidation intermediate 2,6-dimethylheptanoyl carnitine showed lower levels, indicating a high energy requirement in RCC.

The diagnostic accuracy of the identified differential metabolites for RCC vs. healthy controls was further evaluated. A total of 45 metabolites had a potentially useful diagnostic value, with an AUC above 0.7, and 10 metabolites had a good diagnostic value, with an AUC above 0.8 (Table S4). A multivariate ROC curve-based exploratory analysis was performed to achieve a better predictive model (http://www.metaboanalyst.ca/faces/Secure/upload/RocUploadView.xhtml) using a logistic regression algorithm. As a result, a metabolite panel consisting of 9 metabolites as shown in Table S5 showed the best predictive ability. The 10-fold cross validation for the training set achieved an AUC of 0.905 (Figure 2D). The sensitivity and specificity were 0.871 and 0.902, respectively. Further external validation using an independent sample set was performed and achieved good performance with values for AUC, sensitivity and specificity of 0.885, 0.851 and 0.875, respectively, correctly predicting 26 out of 33 RCC patients in the validation set (Figure 2E).

### Distinguishing RCC From Benign Kidney Tumors by Urine Metabolomics

**Figure 3:**
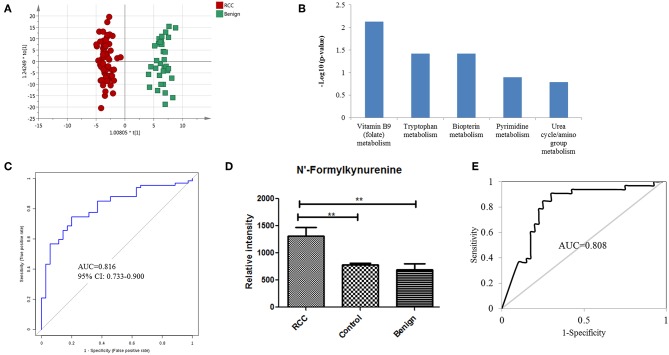
Analysis of urine metabolomic variation between RCC and benign. **(A)** OPLS-DA model based on human urine for classification of RCC and benign in the training set. **(B)** Top five shifted metabolic pathways in RCC compared with benign group. **(C)** ROC plot with 10-fold cross-validation in the training set for distinction of RCC and benign based on metabolites panel of L-3-Hydroxykynurenine, 1,7-Dimethylguanosine, and Tetrahydroaldosterone-3-glucuronide. **(D)** Relative intensity of N-formylkynurenine in RCC, benign and control groups in the training set. **(E)** ROC plot of N-Formylkynurenine for distinction of RCC and non-RCC in the validation group.

Distinguishing RCC from benign kidney tumors was further performed to explore metabolic differences between the two groups. PCA analysis showed slight discrimination of RCC from benign tumors (Figure S4). Furthermore, an OPLS-DA model achieved significant separation (*p* < 0.05) (Figure 3A), with 694 features contributing to group separation (VIP value > 1). However, only 39 features showed a significant *p*-value below 0.05, indicating larger inter-individual variations. Differential features were submitted to perform pathway enrichment analysis using the Mummichog algorithm. Several pathways, including folate metabolism, tryptophan metabolism and biopterin metabolism, were significantly enriched in RCC compared to the benign group (Figure 3B; Table S6).

Further annotation of the top discriminatory features determined by MS/MS evaluation identified 22 significantly differentially detected metabolites (Table S7). ROC analysis showed that all 22 metabolites have a good diagnostic value for RCC and benign controls with AUC values above 0.70 (Table S8). Using a logistic regression algorithm, a metabolite panel consisting of L-3-hydroxykynurenine, 1,7-dimethylguanosine, and tetrahydroaldosterone-3-glucuronide was used to establish a robust model for distinguishing between RCC and benign samples. The AUC was 0.834 for the training dataset and 0.816 for 10-fold cross-validation (Figure 3C). The sensitivity and specificity were 0.741 and 0.794, respectively, for the training set and 0.746 and 0.800, respectively, for cross-validation.

### N-formylkynurenine as a Potential Biomarker for the Differential Diagnosis of RCC vs. Non-RCC

According to the above analysis, urine metabolites could distinguish RCC from healthy and benign controls with high accuracy. Perhaps the common differential metabolites of the two comparisons could be used to diagnose RCC from both healthy and benign controls (non-RCCs). The present results found one common differential metabolite, N'-formylkynurenine, which could discriminate RCC not only from healthy controls but also from benign controls. The relative content of N-formylkynurenine in the RCC group and non-RCC group was plotted in Figure 3D, showing higher levels of N-formylkynurenine in the RCC group compared with the non-RCC group. The relative content of N-formylkynurenine showed a 1.67-fold increase compared to the healthy control group and showed a 2.07-fold increase compared to the benign control group. These results suggest that accumulation of N-formylkynurenine in RCC patients' urine may be used as a potential biomarker for RCC diagnosis.

As a potential biomarker for RCC diagnosis compared to non-RCC cases, we further validated the predictive ability of N-formylkynurenine using the validation group consisting of 33 RCC subjects, 33 healthy controls, and 7 benign controls. The AUC value of the ROC curve was 0.808 (Figure 3E). The sensitivity and specificity were 0.848 and 0.838, respectively. These results suggest that N-formylkynurenine could be significant as a potentially useful metabolite for RCC distinction from healthy controls and benign controls.

### Distinguishing RCC Stages by Urine Metabolomics

Due to RCC sample size limitations, we combined RCC samples of the training set and the validation set. Overall, 84 patients were diagnosed as the pT1 stage, 2 as the pT2 stage, 10 as the pT3 stage, and 4 as the pT4 stage according to pathologic evaluation. T1 and T2 are designated as early stage RCC because the tumor lesion is inside the kidney. T3 and T4 are designated as late stage RCC because the tumor has spread to other organs. Herein, the samples from pT3 and pT4 were relatively few in number, and we performed a pilot comparison of metabolic profiling between early stage and late stage RCC. A PCA score plot showed overlap between early and late stages (Figure S5A). Furthermore, OPLS-DA showed improved separation with R2Y = 0.71 and Q2 = 0.33 (Figure S5B), with 156 features contributing to stage separation. A total of 24 differential metabolites were identified (Table S9), and 12 of them have potential diagnostic value with an AUC above 0.70 (Table S10). A panel consisting of thymidine, cholic acid glucuronide, alanyl-proline, isoleucyl-hydroxyproline, and myristic acid was used for predictive model construction using logistic regression. The AUC value was 0.881 for the testing dataset and 0.813 for the 10-fold cross-validation (Figure S5C). The sensitivity and specificity were 0.921 and 0.756, respectively, for the testing set and 0.857 and 0.721, respectively, for the cross-validation. These results indicate acceptable performance of RCC staging using urine metabolomics.

## Discussion

Our results suggest that the urine metabolome could differentiate RCC patients from healthy controls and from benign kidney tumor patients. Potential biomarkers for RCC and RCC stages were explored and discovered tentatively, which provide new insights into RCC diagnosis. This is the first attempt at applying urine metabolomics for differential diagnosis of RCC vs. benign kidney tumors.

### Metabolite Regulation in RCC Compared With Healthy Controls

Androstenedione, 7-alpha-hydroxy-3-oxochol-4-en-24-oic acid and lithocholyltaurine are the most significantly differentially detected metabolites that have good prediction accuracy between RCC and healthy controls, likely playing important roles during RCC occurrence. Androstendione occurs naturally in the body and is a direct precursor to the hormone testosterone. The conversion of androstendione to testosterone via 17-beta-hydroxysteroid dehydrogenase occurs in the kidney (8). Androstendione levels could be affected by the kidney function status, showing inverse linear associations with renal function (9). Increased secretion levels of androstenedione could result from glomerular filtration function disorder in RCC patients. Lithocholyltaurine and 7-alpha-hydroxy-3-oxochol-4-en-24-oic acid are bile acid conjugation molecules. It has been reported that renal bile acid excretion is a cause of neoplastic lesions (10) and could cause partially reversible renal tubular damage (11). We also noted increases in some dipeptides that may be produced through protein degradation/reutilization processes, such as lysosomal degradation, phagocytosis, endocytosis, pinocytosis, and autophagy (12).

### Metabolite Regulation in RCC Compared With That in Benign Controls

In the clinic, some small renal carcinomas are still difficult to distinguish from benign kidney tumors, and some benign tumors, such as angiomyolipoma, are easily misdiagnosed as malignant tumors. Early and accurate distinction of benign tumors from RCC could reduce the unnecessary treatment for benign tumors. Biopterin metabolism and tryptophan metabolism were found to be disturbed in RCC compared with benign tumors. Disorders of biopterin metabolism in RCC may be accompanied with impaired functioning of tyrosine and tryptophan hydroxylases and the resultant deficiency of tyrosine- and tryptophan-derived monoamine neurotransmitters (13), which is supported by the disturbed tryptophan metabolism suggested by our results. N-formylkynurenine, an intermediate in tryptophan catabolism, was found to be increased in RCC compared with that in benign controls. Accumulation of serum N-formylkynurenine has been reported in patients and experimental animals with renal diseases (14–17). Moreover, patients with chronic kidney disease are permanently exposed to uremic toxins from the kynurenine pathway, which could be mediated by activation of transcription factor aryl hydrocarbon receptor (AhR) (18). These previous results were consistent with our results in the present work, which suggest a significant accumulation of N-formylkynurenine in RCC urine.

### Distinguishing RCC From Non-RCC: Tryptophan Metabolism as a Target Pathway

**Figure 4:**
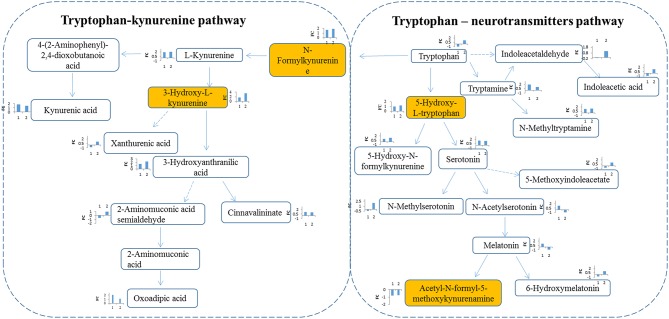
Metabolites interaction in tryptophan metabolism. Tryptophan pathway was significantly changed in RCC. Tryptophan is metabolized through two pathways: tryptophan-kynurenine pathway and tryptophan—neurotransmitters pathway. Metabolites annotation with Mummichog and MS/MS validation are located in colored boxes; Metabolites annotation only with “Mummichog” algorithm are located in blank boxes. The bar figures around each metabolite represents the fold change of metabolite in RCC compared with health control (x-coordinate:1) and the benigns (x-coordinate: 2). Direction of bars represent up-regulated (above X-aixs) or down-regulated (below X-axis).

Tryptophan-kynurenine pathway (KP) metabolites, N-formylkynurenine, kynurenine and 3-hydroxy-L-kynurenine were found to be disturbed in RCC (Figure 4), indicating a potential role of tryptophan metabolism during RCC development. Tryptophan-KP metabolism in cancer has increasingly been recognized as an important microenvironmental factor that suppresses antitumor immune responses (19–21). Depletion of tryptophan induces signaling events in T cells, leading to anergy, apoptosis, and active immunomodulation by accumulating tryptophan-KP metabolites (22). During RCC development, tryptophan-KP metabolism has been found to be highly represented in tissues, which is associated with immune suppression (23). Indoleamine-2,3-dioxygenase (IDO) can possibly catalyze an early step in tryptophan metabolism, regulating the conversion of tryptophan to immunosuppressive metabolites that could work to the tumor's advantage (24). The same has occurred in RCC patient serum and urine (3, 25), which is consistent with our results.

In humans, >95% of tryptophan is metabolized through the kynurenine pathway, and other tryptophan is converted into the key neurotransmitters serotonin and tryptamine (26). Herein, apart from tryptophan-KP metabolite variations, we also observed increase of tryptophan-neurotransmitter metabolites, such as 5-hydroxy-L-tryptophan, serotonin, and tryptamine, in RCC (Figure 4), indicating neuroendocrine involvement of tryptophan metabolism in human RCC (27). Neuroendocrine markers have been reported in RCC serum, including chromogranin (Cg) A and B, pancreastatin and serotonin (28). These results indicate the potential value of tryptophan metabolism for designing new targets of RCC.

## Conclusions

In conclusion, we have applied urine metabolomic approach to dissect metabolic features of RCC not only compared with healthy controls but also compared with benign controls. These results showed markedly different metabolic profiles between RCC and healthy controls or benign controls, which suggests the feasibility of utilizing urine metabolites for early clinical diagnosis. Potential biomarkers for RCC were tentatively explored. The changes in tryptophan metabolism have profound implications for designing new targets for RCC. Moreover, N-formylkynurenine was discovered to have potential value for RCC distinction from healthy and benign controls. However, in our present study, the influence of diet on urine metabolomics might not be completely eliminated, though all subjects were from the same region. For future validation analysis, the influence of diet would be systematically analyzed and evaluated using a diet standardization design.

## Ethics Statement

This study was approved by the Institutional Review Board of the Institute of Basic Medical Sciences, Chinese Academy of Medical Sciences. All human subjects provided informed consent before participating in this study.

## Author Contributions

XoL, MZ, XnL, WS, and YZ conceived and designed the study, helped to interpret the data, wrote the first draft of the manuscript, and contributed to the final version of the manuscript. MZ, HL, and ZW collected the urine samples. HS and XT helped to interpret the data, performed the statistical analysis, and contributed to the final version of the manuscript. ZG and JL helped the data analysis. WS is the guarantor of this work and, as such, had full access to all the data in the study and takes responsibility for the integrity of the data and the accuracy of the data analysis.

### Conflict of Interest Statement

The authors declare that the research was conducted in the absence of any commercial or financial relationships that could be construed as a potential conflict of interest.

## Abbreviations

AUC: area under the curve
BMI: body mass index
CT: computed tomography
IDO: indoleamine-2,3-dioxygenase
KP: kynurenine pathway
LC-MS: liquid chromatography—mass spectrometry
NMR: nuclear magnetic resonance
OPLS-DA: orthogonal partial least squares discriminant analysis
PCA: principal component analysis
QC: quality control
RCC: renal cell carcinoma.
