# Supplementary material for: Urine Metabolomics for Renal Cell Carcinoma (RCC) Prediction: Tryptophan Metabolism as an Important Pathway in RCC
Source: Front Oncol. 2019 Jul 17;9:663. doi: 10.3389/fonc.2019.00663 (PMC6653643; doi:10.3389/fonc.2019.00663)
Supplement: Supplementary file 1 [file Presentation_1.PPTX]

## Slide 1
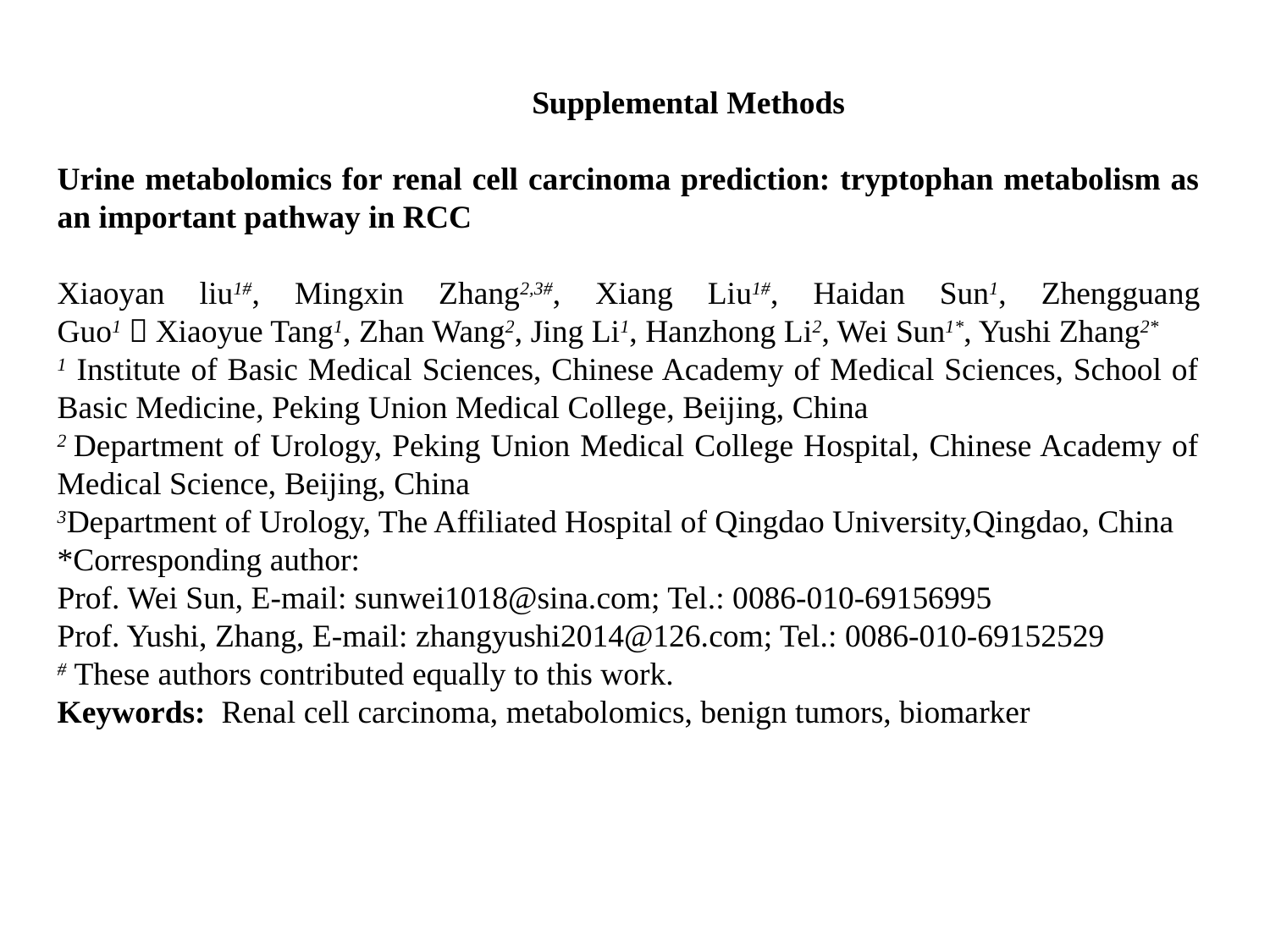

Supplemental Methods
Urine metabolomics for renal cell carcinoma prediction: tryptophan metabolism as an important pathway in RCC
Xiaoyan liu1#, Mingxin Zhang2,3#, Xiang Liu1#, Haidan Sun1, Zhengguang Guo1，Xiaoyue Tang1, Zhan Wang2, Jing Li1, Hanzhong Li2, Wei Sun1*, Yushi Zhang2*
1 Institute of Basic Medical Sciences, Chinese Academy of Medical Sciences, School of Basic Medicine, Peking Union Medical College, Beijing, China
2 Department of Urology, Peking Union Medical College Hospital, Chinese Academy of Medical Science, Beijing, China
3Department of Urology, The Affiliated Hospital of Qingdao University,Qingdao, China
*Corresponding author:
Prof. Wei Sun, E-mail: sunwei1018@sina.com; Tel.: 0086-010-69156995
Prof. Yushi, Zhang, E-mail: zhangyushi2014@126.com; Tel.: 0086-010-69152529
# These authors contributed equally to this work.
Keywords: Renal cell carcinoma, metabolomics, benign tumors, biomarker

## Slide 2
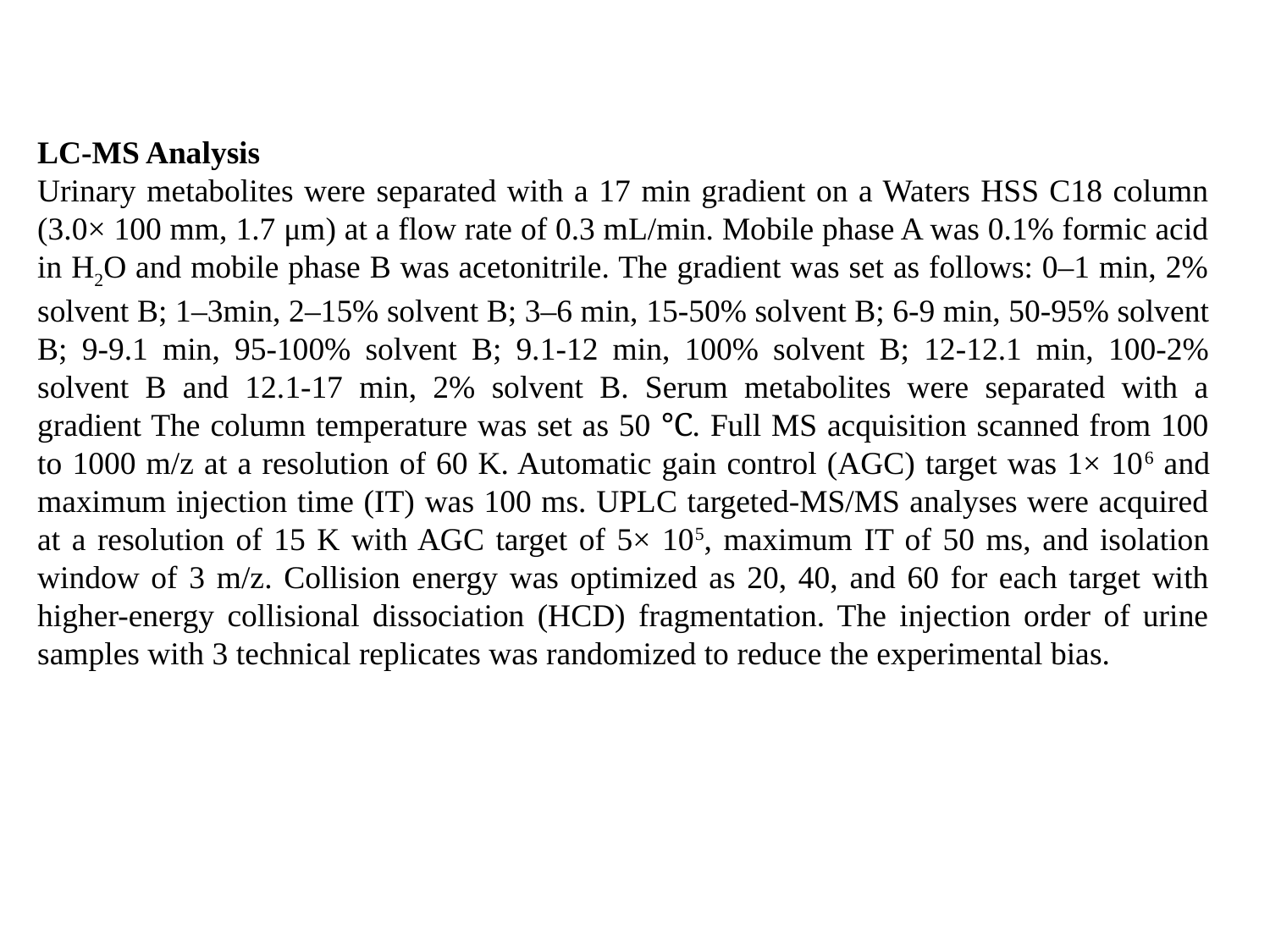

LC-MS Analysis
Urinary metabolites were separated with a 17 min gradient on a Waters HSS C18 column (3.0× 100 mm, 1.7 μm) at a flow rate of 0.3 mL/min. Mobile phase A was 0.1% formic acid in H2O and mobile phase B was acetonitrile. The gradient was set as follows: 0–1 min, 2% solvent B; 1–3min, 2–15% solvent B; 3–6 min, 15-50% solvent B; 6-9 min, 50-95% solvent B; 9-9.1 min, 95-100% solvent B; 9.1-12 min, 100% solvent B; 12-12.1 min, 100-2% solvent B and 12.1-17 min, 2% solvent B. Serum metabolites were separated with a gradient The column temperature was set as 50 ℃. Full MS acquisition scanned from 100 to 1000 m/z at a resolution of 60 K. Automatic gain control (AGC) target was 1× 10^6^ and maximum injection time (IT) was 100 ms. UPLC targeted-MS/MS analyses were acquired at a resolution of 15 K with AGC target of 5× 10^5^, maximum IT of 50 ms, and isolation window of 3 m/z. Collision energy was optimized as 20, 40, and 60 for each target with higher-energy collisional dissociation (HCD) fragmentation. The injection order of urine samples with 3 technical replicates was randomized to reduce the experimental bias.

## Slide 3
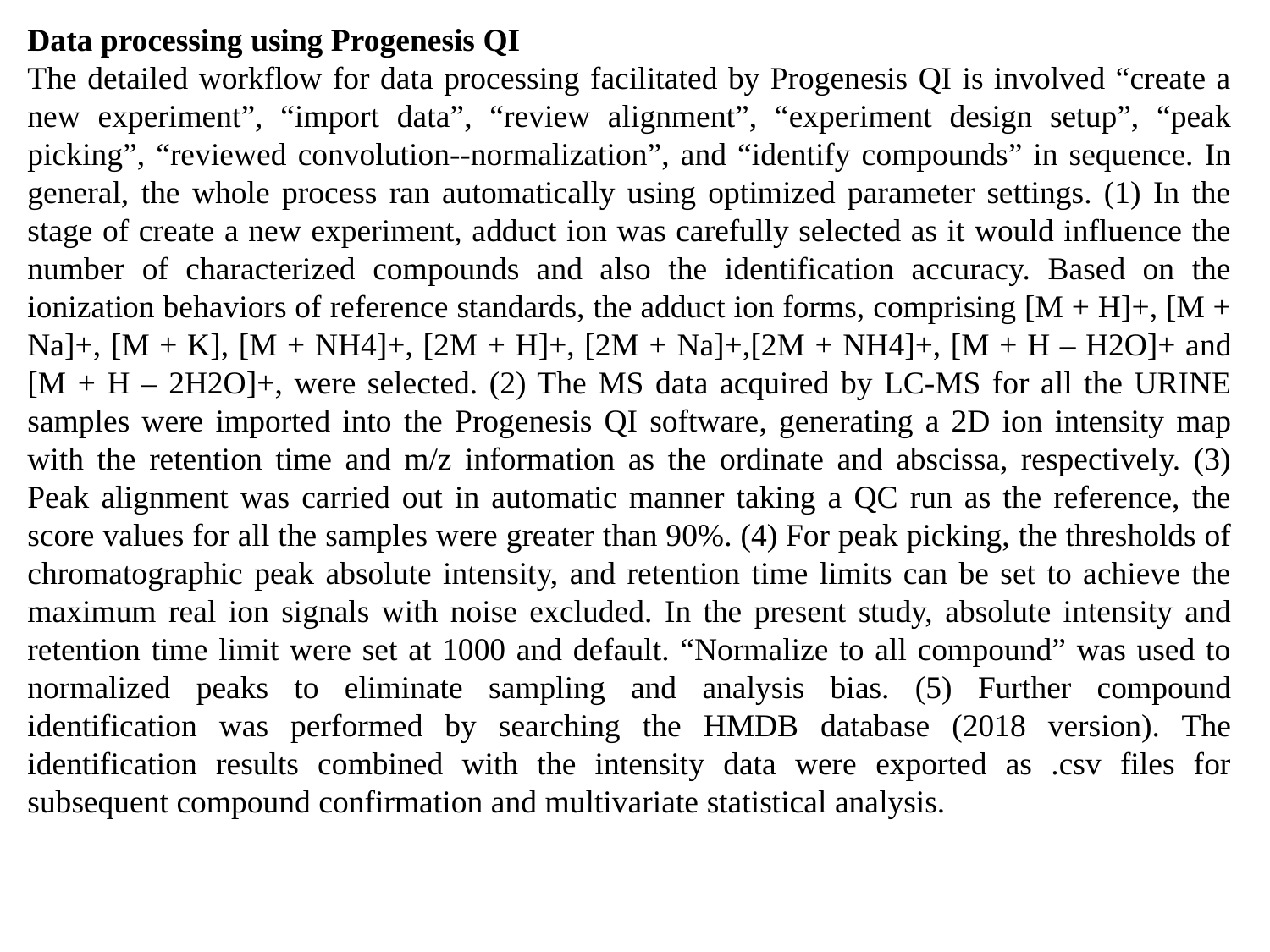

Data processing using Progenesis QI
The detailed workflow for data processing facilitated by Progenesis QI is involved “create a new experiment”, “import data”, “review alignment”, “experiment design setup”, “peak picking”, “reviewed convolution--normalization”, and “identify compounds” in sequence. In general, the whole process ran automatically using optimized parameter settings. (1) In the stage of create a new experiment, adduct ion was carefully selected as it would influence the number of characterized compounds and also the identification accuracy. Based on the ionization behaviors of reference standards, the adduct ion forms, comprising [M + H]+, [M + Na]+, [M + K], [M + NH4]+, [2M + H]+, [2M + Na]+,[2M + NH4]+, [M + H – H2O]+ and [M + H – 2H2O]+, were selected. (2) The MS data acquired by LC-MS for all the URINE samples were imported into the Progenesis QI software, generating a 2D ion intensity map with the retention time and m/z information as the ordinate and abscissa, respectively. (3) Peak alignment was carried out in automatic manner taking a QC run as the reference, the score values for all the samples were greater than 90%. (4) For peak picking, the thresholds of chromatographic peak absolute intensity, and retention time limits can be set to achieve the maximum real ion signals with noise excluded. In the present study, absolute intensity and retention time limit were set at 1000 and default. “Normalize to all compound” was used to normalized peaks to eliminate sampling and analysis bias. (5) Further compound identification was performed by searching the HMDB database (2018 version). The identification results combined with the intensity data were exported as .csv files for subsequent compound confirmation and multivariate statistical analysis.

## Slide 4
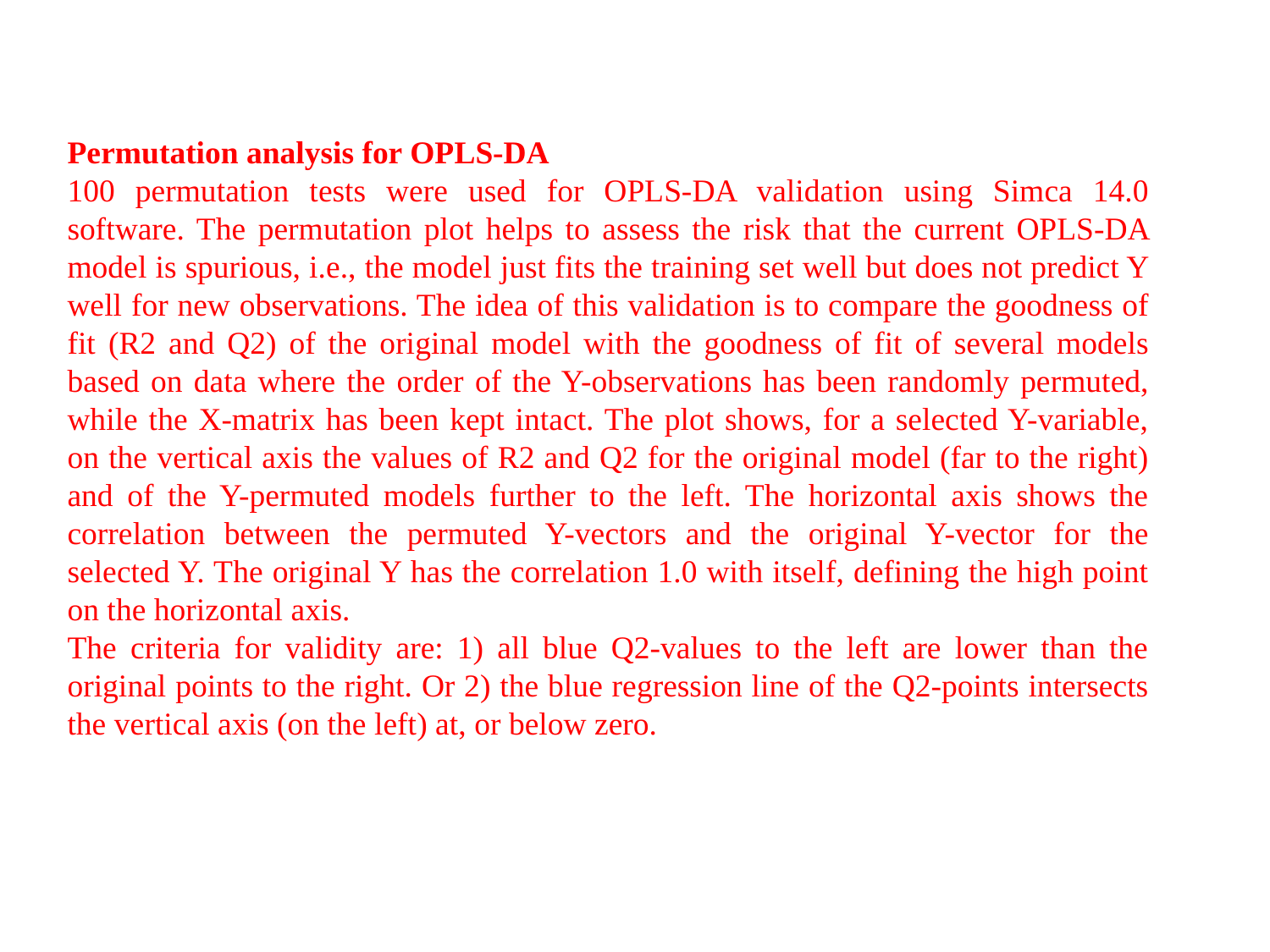

Permutation analysis for OPLS-DA
100 permutation tests were used for OPLS-DA validation using Simca 14.0 software. The permutation plot helps to assess the risk that the current OPLS-DA model is spurious, i.e., the model just fits the training set well but does not predict Y well for new observations. The idea of this validation is to compare the goodness of fit (R2 and Q2) of the original model with the goodness of fit of several models based on data where the order of the Y-observations has been randomly permuted, while the X-matrix has been kept intact. The plot shows, for a selected Y-variable, on the vertical axis the values of R2 and Q2 for the original model (far to the right) and of the Y-permuted models further to the left. The horizontal axis shows the correlation between the permuted Y-vectors and the original Y-vector for the selected Y. The original Y has the correlation 1.0 with itself, defining the high point on the horizontal axis.
The criteria for validity are: 1) all blue Q2-values to the left are lower than the original points to the right. Or 2) the blue regression line of the Q2-points intersects the vertical axis (on the left) at, or below zero.

## Slide 5
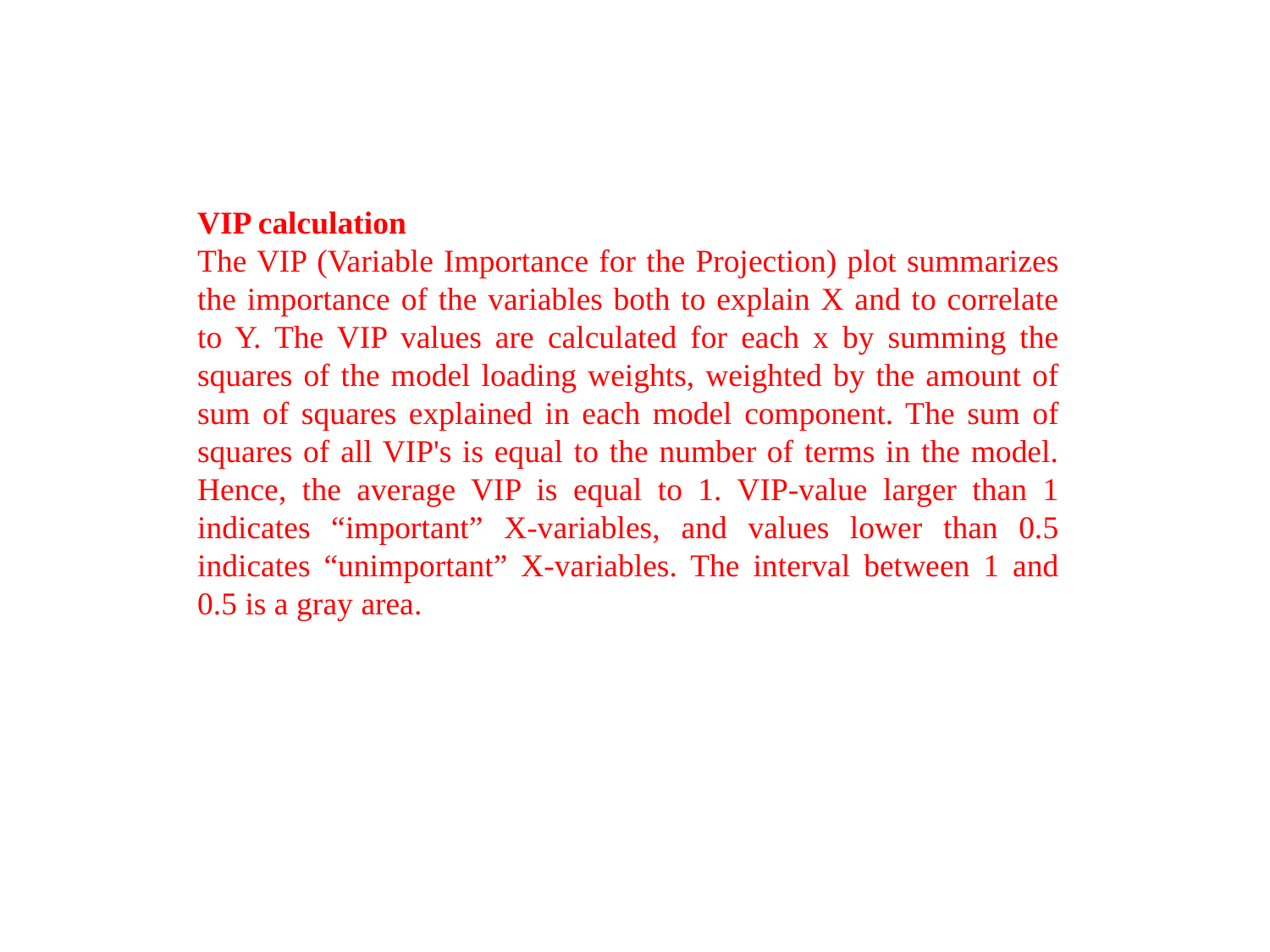

VIP calculation
The VIP (Variable Importance for the Projection) plot summarizes the importance of the variables both to explain X and to correlate to Y. The VIP values are calculated for each x by summing the squares of the model loading weights, weighted by the amount of sum of squares explained in each model component. The sum of squares of all VIP's is equal to the number of terms in the model. Hence, the average VIP is equal to 1. VIP-value larger than 1 indicates “important” X-variables, and values lower than 0.5 indicates “unimportant” X-variables. The interval between 1 and 0.5 is a gray area.

## Slide 6
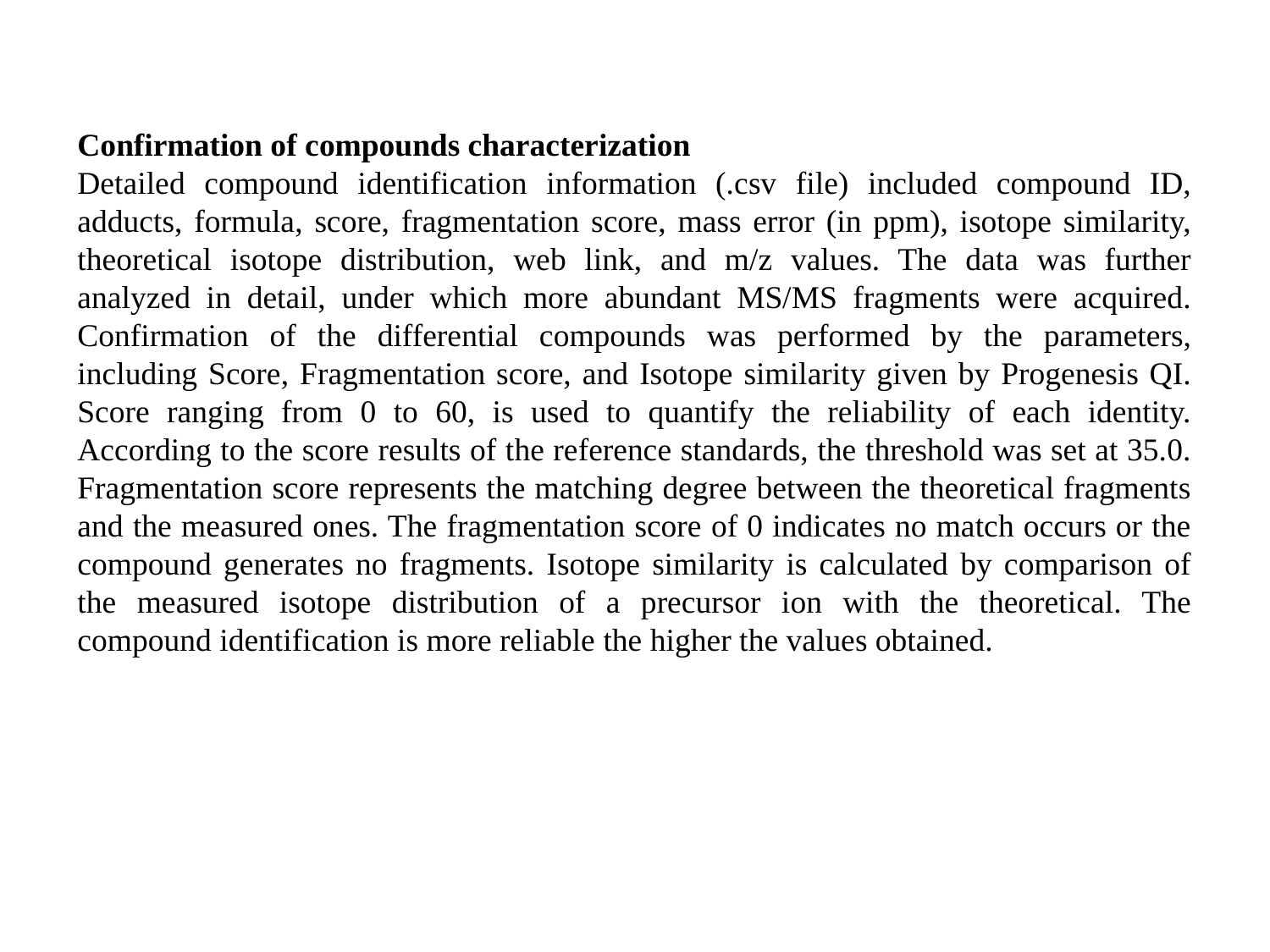

Confirmation of compounds characterization
Detailed compound identification information (.csv file) included compound ID, adducts, formula, score, fragmentation score, mass error (in ppm), isotope similarity, theoretical isotope distribution, web link, and m/z values. The data was further analyzed in detail, under which more abundant MS/MS fragments were acquired. Confirmation of the differential compounds was performed by the parameters, including Score, Fragmentation score, and Isotope similarity given by Progenesis QI. Score ranging from 0 to 60, is used to quantify the reliability of each identity. According to the score results of the reference standards, the threshold was set at 35.0. Fragmentation score represents the matching degree between the theoretical fragments and the measured ones. The fragmentation score of 0 indicates no match occurs or the compound generates no fragments. Isotope similarity is calculated by comparison of the measured isotope distribution of a precursor ion with the theoretical. The compound identification is more reliable the higher the values obtained.

## Slide 7
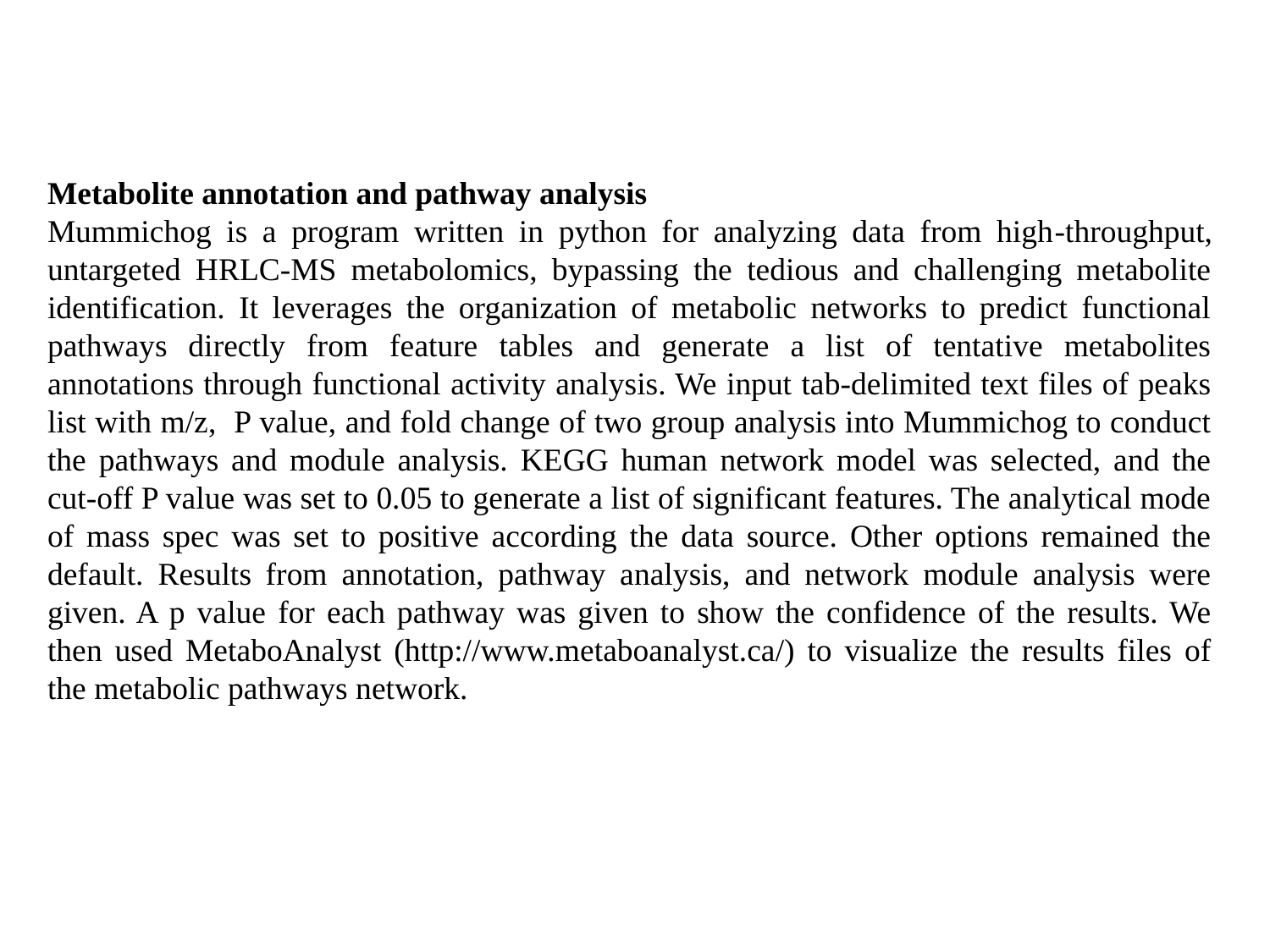

Metabolite annotation and pathway analysis
Mummichog is a program written in python for analyzing data from high‐throughput, untargeted HRLC‐MS metabolomics, bypassing the tedious and challenging metabolite identification. It leverages the organization of metabolic networks to predict functional pathways directly from feature tables and generate a list of tentative metabolites annotations through functional activity analysis. We input tab‐delimited text files of peaks list with m/z, P value, and fold change of two group analysis into Mummichog to conduct the pathways and module analysis. KEGG human network model was selected, and the cut‐off P value was set to 0.05 to generate a list of significant features. The analytical mode of mass spec was set to positive according the data source. Other options remained the default. Results from annotation, pathway analysis, and network module analysis were given. A p value for each pathway was given to show the confidence of the results. We then used MetaboAnalyst (http://www.metaboanalyst.ca/) to visualize the results files of the metabolic pathways network.
