# Supplementary material for: Urine Metabolomics for Renal Cell Carcinoma (RCC) Prediction: Tryptophan Metabolism as an Important Pathway in RCC
Source: Front Oncol. 2019 Jul 17;9:663. doi: 10.3389/fonc.2019.00663 (PMC6653643; doi:10.3389/fonc.2019.00663)

**Supplemental Figures**

**Urine metabolomics for renal cell carcinoma prediction: tryptophan metabolism as an important pathway in RCC**

Xiaoyan liu*^1#^*, mingxin Zhang*^2,3#^*, Xiang Liu*^1#^*, Haidan Sun*^1^*, Zhengguang Guo*^1^*，Xiaoyue Tang*^1^*, Zhan Wang*^2^*, Jing Li*^1^*, Hanzhong Li*^2^*, Wei Sun*^1*^*, Yushi Zhang*^2*^*

*^1^* Institute of Basic Medical Sciences, Chinese Academy of Medical Sciences, School of Basic Medicine, Peking Union Medical College, Beijing, China

*^2^* Department of Urology, Peking Union Medical College Hospital, Chinese Academy of Medical Science, Beijing, China

*^3^*Department of Urology, The Affiliated Hospital of Qingdao University,Qingdao, China

*Corresponding author:

Prof. Wei Sun, E-mail: sunwei1018@sina.com; Tel.: 0086-010-69156995

Prof. Yushi, Zhang, E-mail: zhangyushi2014@126.com; Tel.: 0086-010-69152529

*^#^* These authors contributed equally to this work.

**Keywords:** Renal cell carcinoma, metabolomics, benign tumors, biomarker

Fig. S1.Stability assessment of analysis platform. The PCA score plot showed the cluster of samples and QCs. Tight cluster of QC samples indicated good stability of the analysis process. Blue, samples; Yellow, QCs.


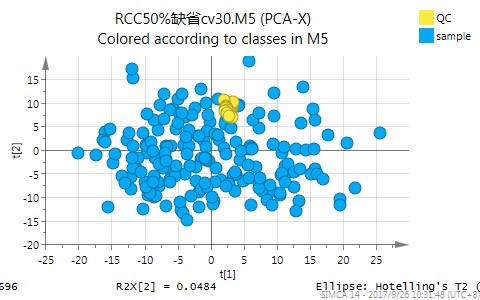


Fig. S2. 100 permutation tests of the OPLS-DA model based on RCC and control urine metabolomics. All blue Q2-values to the left are lower than the original points to the right. And the blue regression line of the Q2-points intersects the vertical axis (on the left), indicated no over fitting of OPLS-DA model.


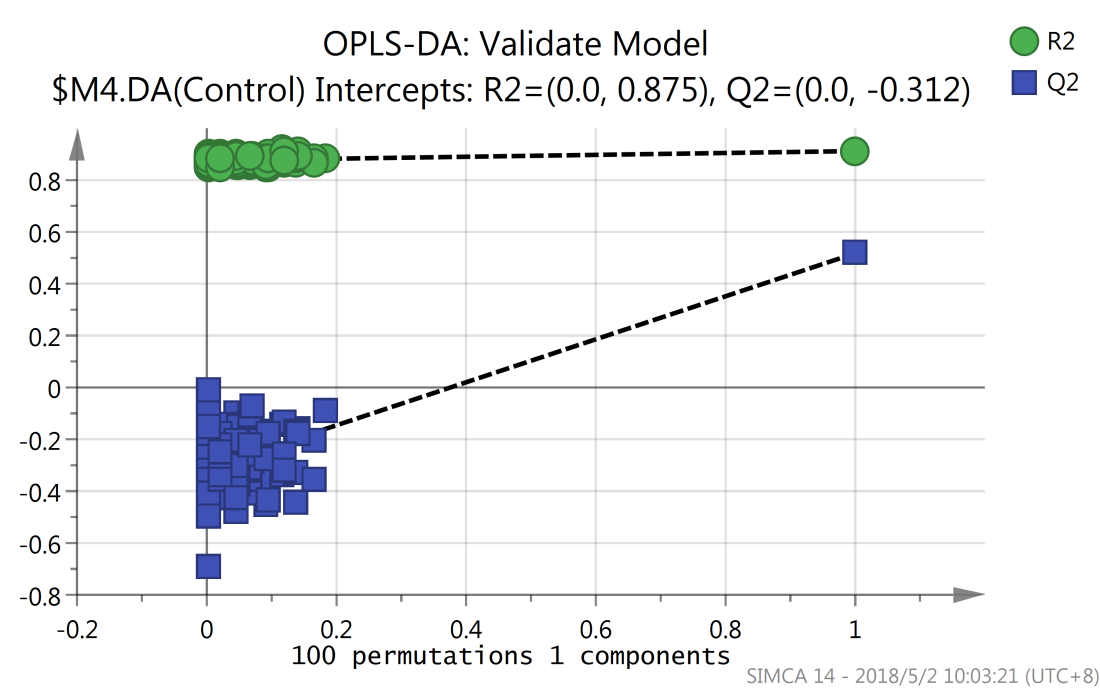


Fig. S3. Predicted activity network in RCC compared with controls. The pathway enrichment was performed using Mummichog algorithm based on MetaboAnalyst 3.0 platform (See Supplementary method). The marked pathway indicated confidential pathways with p < 0.05.


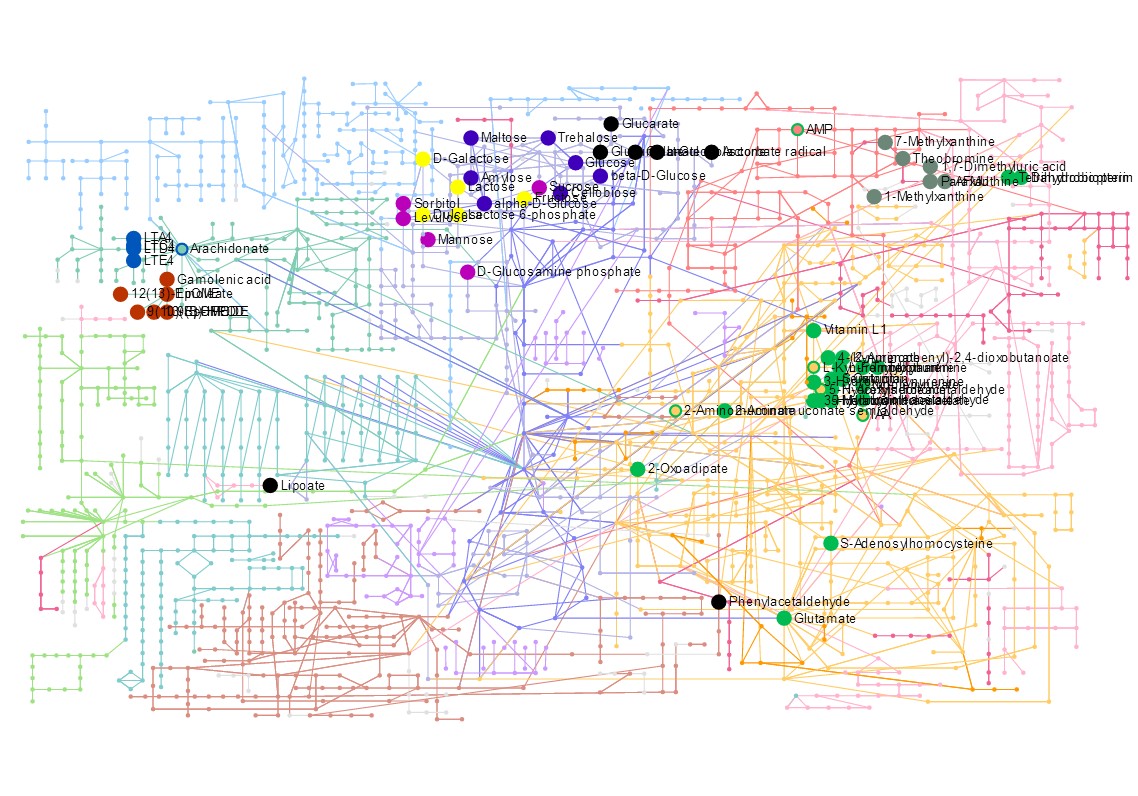


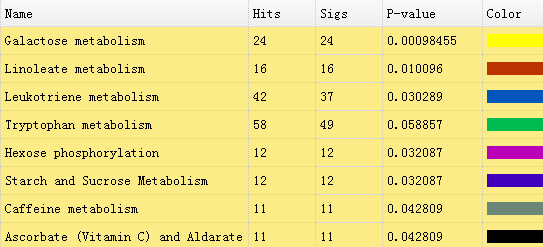

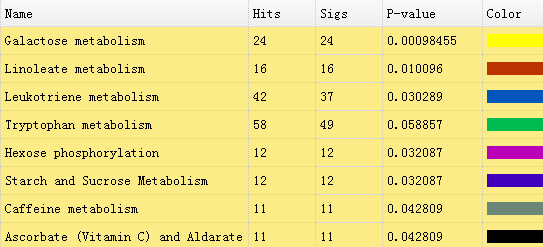


Fig. S4. Score plot of unsupervised PCA overview of urinary metabolic profiling between RCC and benign. Slight separation was observed.


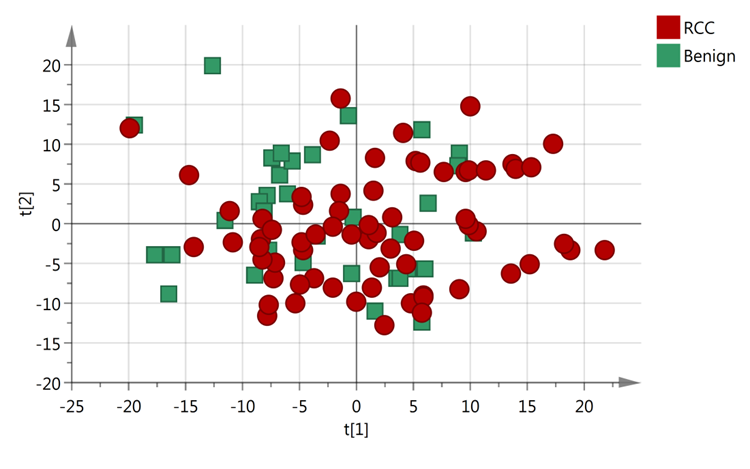


Fig. S5. Multivariate analysis of urine metabolomics between early and late RCC. a. Score plot of unsupervised PCA overview of urinary metabolic profiling between early- and late-RCC. b. Score plot of OPLS-DA model based on urine for stages classification. c. ROC plot with 10-fold cross-validation for distinction of RCC stages based on metabolites panel of thymidine, cholic acid glucuronide, alanyl-proline, isoleucyl-hydroxyproline and myristic acid, achieved an AUC value of 0.813.


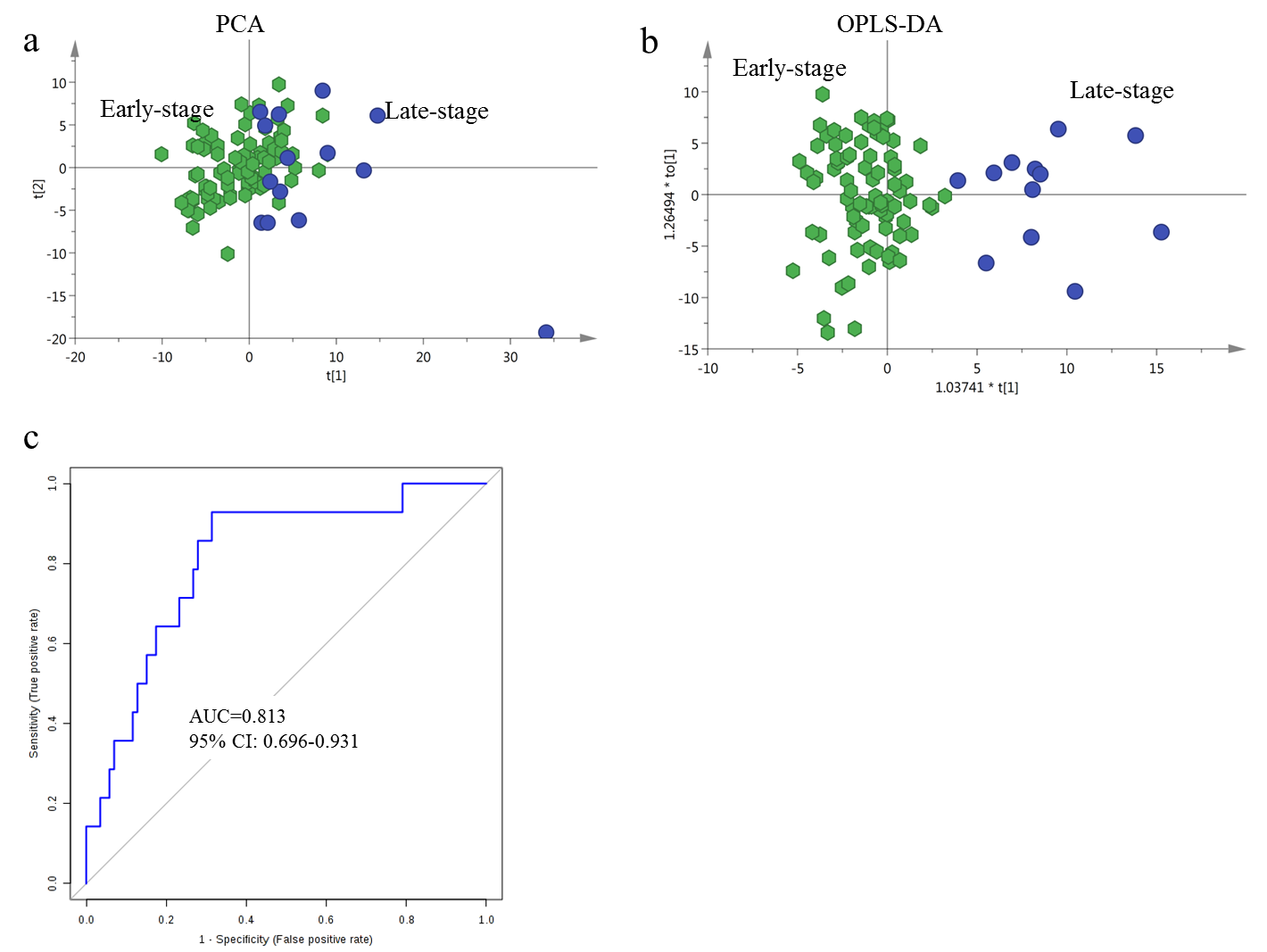

Supplement: Supplementary file 2 [file Data_Sheet_1.docx]
