# Supplementary material for: Urine Metabolomics for Renal Cell Carcinoma (RCC) Prediction: Tryptophan Metabolism as an Important Pathway in RCC
Source: Front Oncol. 2019 Jul 17;9:663. doi: 10.3389/fonc.2019.00663 (PMC6653643; doi:10.3389/fonc.2019.00663)
Supplement: Supplementary file 3 [file Data_Sheet_2.docx]

**Supplemental Tables**

**Urine metabolomics for renal cell carcinoma prediction: tryptophan metabolism as an important pathway in RCC**

Xiaoyan liu*^1#^*, Mingxin Zhang*^2,3#^*, Xiang Liu*^1#^*, Haidan Sun*^1^*, Zhengguang Guo*^1^*，Xiaoyue Tang*^1^*, Zhan Wang*^2^*, Jing Li*^1^*, Hanzhong Li*^2^*, Wei Sun*^1*^*, Yushi Zhang*^2*^*

*^1^* Institute of Basic Medical Sciences, Chinese Academy of Medical Sciences, School of Basic Medicine, Peking Union Medical College, Beijing, China

*^2^* Department of Urology, Peking Union Medical College Hospital, Chinese Academy of Medical Science, Beijing, China

*^3^*Department of Urology, The Affiliated Hospital of Qingdao University,Qingdao, China

*Corresponding author: Prof. Wei Sun, E-mail: sunwei1018@sina.com; Tel.: 0086-010-69156995

Prof. Yushi, Zhang, E-mail: zhangyushi2014@126.com; Tel.: 0086-010-69152529

*^#^* These authors contributed equally to this work.

**Keywords:** Renal cell carcinoma, metabolomics, benign tumors, biomarker

**Table S1** Detailed information of all enrolled subjects

| **RCC-training set** | | | | | | | | | |
| --- | --- | --- | --- | --- | --- | --- | --- | --- | --- |
| **ID** | **Sex** | **Age** | **Pathology** | **Pathological stage** | **AJCC stage** | **eGFR （ml/min/1.7312）** | **BMI** | **Urine leukocyte** | **Urine erythrocyte** |
| R1016 | F | 43 | Clear cell renal cell carcinoma | T1b | 1 | 100.99 | 27.10 | - | - |
| R1054 | F | 45 | Clear cell renal cell carcinoma | T1b | 1 | 99.91 | 22.10 | - | - |
| R993 | F | 55 | Clear cell renal cell carcinoma | T3a | 3 | 85.92 | 23.03 | - | - |
| R926 | F | 56 | Clear cell renal cell carcinoma | T3a | 3 | 86.46 | 24.22 | - | - |
| R878 | F | 59 | Clear cell renal cell carcinoma | T1a | 1 | 88.13 | 19.71 | - | - |
| R913 | F | 66 | Clear cell renal cell carcinoma | T1a | 1 | 82.34 | 25.00 | - | - |
| R874 | F | 69 | Clear cell renal cell carcinoma | T1a | 1 | 87.50 | 24.61 | - | - |
| R964 | F | 71 | Papillary renal cell carcinoma | T1a | 1 | 89.97 | 23.05 | - | - |
| R934 | F | 78 | Clear cell renal cell carcinoma | T3a | 3 | 89.39 | 25.18 | - | - |
| R995 | F | 68 | Clear cell renal cell carcinoma | T1a | 1 | 90.03 | 24.56 | - | - |
| R933 | F | 40 | Clear cell renal cell carcinoma | T1a | 1 | 94.60 | 20.94 | - | - |
| R1024 | F | 61 | Chromophobe cell carcinoma | T1b | 1 | 95.84 | 23.95 | - | - |
| R1074 | F | 33 | Chromophobe cell carcinoma | T1b | 1 | 98.37 | 21.23 | - | - |
| R994 | F | 15 | Clear cell renal cell carcinoma | T3a | 3 | 103.60 | 17.63 | - | - |
| R965 | F | 72 | Clear cell renal cell carcinoma | T1a | 1 | 105.62 | 30.86 | - | - |
| R1006 | F | 67 | Clear cell renal cell carcinoma | T3a | 1 | 107.18 | 28.73 | - | - |
| R1094 | F | 44 | Clear cell renal cell carcinoma | T1b | 1 | 109.14 | 26.95 | - | - |
| R924 | F | 28 | Clear cell renal cell carcinoma | T1b | 1 | 112.26 | 19.49 | - | - |
| R1104 | F | 64 | Clear cell renal cell carcinoma | T1a | 1 | 116.18 | 24.65 | - | - |
| R889 | M | 33 | Papillary renal cell carcinoma | T1b | 1 | 116.21 | 25.82 | - | - |
| R1086 | M | 44 | Papillary renal cell carcinoma | T3a | 4 | 98.37 | 23.53 | - | - |
| R1110 | M | 44 | Clear cell renal cell carcinoma | T1a | 1 | 99.04 | 35.66 | - | - |
| R967 | M | 44 | Clear cell renal cell carcinoma | T1a | 1 | 99.54 | 25.03 | - | - |
| R1084 | M | 47 | Clear cell renal cell carcinoma | T1a | 1 | 92.74 | 22.68 | - | - |
| R951 | M | 47 | Clear cell renal cell carcinoma | T3a | 3 | 94.13 | 21.88 | - | - |
| R996 | M | 47 | Clear cell renal cell carcinoma | T3a | 3 | 94.60 | 21.88 | - | - |
| R972 | M | 48 | Papillary renal cell carcinoma | T1a | 1 | 95.84 | 23.60 | - | - |
| R917 | M | 48 | Clear cell renal cell carcinoma | T1a | 1 | 96.92 | 27.68 | - | TRACE |
| R980 | M | 51 | Clear cell renal cell carcinoma | T1b | 1 | 89.97 | 27.44 | - | - |
| R891 | M | 51 | Clear cell renal cell carcinoma | T3a | 3 | 90.03 | 23.03 | - | - |
| R936 | M | 52 | Clear cell renal cell carcinoma | T1a | 1 | 90.72 | 24.00 | - | - |
| R986 | M | 56 | Clear cell renal cell carcinoma | T1b | 1 | 87.50 | 20.68 | - | - |
| R944 | M | 58 | Clear cell renal cell carcinoma | T1b | 1 | 81.40 | 26.30 | - | - |
| R871 | M | 59 | Clear cell renal cell carcinoma | T1b | 1 | 82.25 | 23.15 | - | - |
| R1020 | M | 62 | Clear cell renal cell carcinoma | T3a | 3 | 83.23 | 21.47 | - | - |
| R971 | M | 69 | Clear cell renal cell carcinoma | T1a | 1 | 86.46 | 27.72 | - | - |
| R952 | M | 73 | Clear cell renal cell carcinoma | T3a | 3 | 87.70 | 26.22 | - | - |
| R1066 | M | 75 | Clear cell renal cell carcinoma | T1a | 1 | 88.13 | 24.57 | - | - |
| R970 | M | 82 | Clear cell renal cell carcinoma | T1a | 1 | 89.39 | 26.06 | - | - |
| R1015 | M | 63 | Clear cell renal cell carcinoma | T1b | 1 | 85.92 | 26.70 | - | - |
| R1058 | M | 53 | Clear cell renal cell carcinoma | T1a | 1 | 89.97 | 29.76 | - | - |
| R872 | M | 38 | Clear cell renal cell carcinoma | T1b | 1 | 87.50 | 26.25 | - | - |
| R881 | M | 71 | Clear cell renal cell carcinoma | T3a | 3 | 87.70 | 24.24 | - | - |
| R954 | M | 42 | Clear cell renal cell carcinoma | T1a | 1 | 88.13 | 27.43 | - | - |
| R1057 | M | 64 | Clear cell renal cell carcinoma | T1b | 1 | 88.22 | 28.69 | - | - |
| R914 | M | 60 | Clear cell renal cell carcinoma | T1b | 1 | 89.39 | 28.41 | - | - |
| R1109 | M | 54 | Clear cell renal cell carcinoma | T1b | 1 | 89.97 | 24.90 | - | - |
| R947 | M | 54 | Clear cell renal cell carcinoma | T1b | 4 | 89.97 | 38.10 | - | - |
| R882 | M | 39 | Clear cell renal cell carcinoma | T1a | 1 | 90.72 | 26.12 | - | - |
| R1095 | M | 35 | Clear cell renal cell carcinoma | T1a | 1 | 92.74 | 25.86 | - | - |
| R946 | M | 54 | Clear cell renal cell carcinoma | T1a | 1 | 94.13 | 27.85 | - | - |
| R1079 | M | 59 | Clear cell renal cell carcinoma | T1b | 1 | 96.92 | 29.00 | - | - |
| R1114 | M | 39 | Clear cell renal cell carcinoma | T1a | 1 | 99.04 | 23.39 | - | - |
| R1033 | M | 56 | Clear cell renal cell carcinoma | T1a | 1 | 99.54 | 25.95 | - | - |
| R1099 | M | 55 | Clear cell renal cell carcinoma | T1b | 1 | 99.91 | 24.09 | - | - |
| R1063 | M | 27 | Clear cell renal cell carcinoma | T1a | 1 | 102.05 | 22.37 | - | - |
| R908 | M | 45 | Clear cell renal cell carcinoma | T1b | 1 | 107.55 | 29.41 | - | - |
| R975 | M | 73 | Clear cell renal cell carcinoma | T3a | 3 | 110.30 | 17.99 | - | - |
| R973 | M | 45 | Clear cell renal cell carcinoma | T1a | 1 | 111.26 | 23.67 | - | - |
| R1088 | M | 53 | Clear cell renal cell carcinoma | T1a | 1 | 113.47 | 20.57 | - | - |
| R898 | M | 53 | Clear cell renal cell carcinoma | T1a | 1 | 113.47 | 20.66 | - | - |
| R992 | M | 61 | Clear cell renal cell carcinoma | T1a | 1 | 114.39 | 19.82 | - | - |
| R939 | M | 77 | Clear cell renal cell carcinoma | T1a | 1 | 115.54 | 20.76 | - | - |
| R1096 | M | 68 | Clear cell renal cell carcinoma | T1b | 1 | 116.21 | 25.38 | - | - |
| R890 | M | 47 | Clear cell renal cell carcinoma | T1a | 1 | 135.64 | 25.95 | - | - |
| R1098 | M | 14 | Clear cell renal cell carcinoma | T1b | 1 | 163.31 | 17.65 | - | - |
| R942 | M | 60 | Clear cell renal cell carcinoma | T1a | 1 | 120.05 | 23.89 | - | - |
| **RCC-validation set** | | | | | | | | | |
| R1186 | M | 64 | Clear cell renal cell carcinoma | T1b | 1 | 86.79048 | 23.78121 | - | - |
| R1165 | M | 52 | Clear cell renal cell carcinoma | T1a | 1 | 91.34348 | 22.83737 | - | - |
| R1119 | M | 55 | Clear cell renal cell carcinoma | T1a | 1 | 91.39327 | 29.06977 | - | - |
| R1142 | M | 48 | Clear cell renal cell carcinoma | T1a | 1 | 93.39373 | 27.71769 | - | - |
| R1162 | M | 60 | Clear cell renal cell carcinoma | T1b | 1 | 97.63795 | 25.88057 | - | - |
| R1147 | M | 55 | Clear cell renal cell carcinoma | T1a | 1 | 101.171 | 28.40055 | - | - |
| R1136 | M | 65 | Clear cell renal cell carcinoma | T1b | 1 | 104.1505 | 28.71972 | - | - |
| R1161 | F | 82 | Clear cell renal cell carcinoma | T1a | 1 | 104.9831 | 24.88747 | - | - |
| R1210 | F | 33 | Clear cell renal cell carcinoma | T1a | 1 | 105.3316 | 25.76571 | - | - |
| R1118 | M | 60 | Clear cell renal cell carcinoma | T1b | 1 | 105.529 | 26.07897 | - | - |
| R1182 | M | 65 | Papillary renal cell carcinoma | T1a | 1 | 89.28195 | 21.22449 | - | - |
| R1194 | M | 24 | Clear cell renal cell carcinoma | T1a | 1 | 89.92185 | 25.72756 | - | - |
| R1143 | M | 46 | Papillary renal cell carcinoma | T1a | 1 | 90.26893 | 27.99474 | - | - |
| R1196 | F | 50 | Clear cell renal cell carcinoma | T1a | 1 | 90.41463 | 22.47659 | - | - |
| R1168 | M | 51 | Clear cell renal cell carcinoma | T1a | 1 | 92.38403 | 23.1405 | - | - |
| R1128 | M | 56 | Clear cell renal cell carcinoma | T1a | 1 | 93.44178 | 32.17993 | - | - |
| R1124 | M | 32 | Clear cell renal cell carcinoma | T1b | 1 | 94.44029 | 23.5102 | - | - |
| R1171 | M | 66 | Clear cell renal cell carcinoma | T1b | 1 | 94.73596 | 27.63605 | - | - |
| R1138 | M | 35 | Clear cell renal cell carcinoma | T1b | 1 | 99.72128 | 30.21886 | - | - |
| R1139 | M | 24 | Clear cell renal cell carcinoma | T1a | 1 | 100.1198 | 23.14815 | - | - |
| R1132 | M | 43 | Clear cell renal cell carcinoma | T1a | 1 | 100.1282 | 22.91303 | - | - |
| R1148 | M | 45 | Clear cell renal cell carcinoma | T1a | 1 | 105.7788 | 28.4055 | - | - |
| R1164 | M | 35 | Clear cell renal cell carcinoma | T1a | 1 | 107.7548 | 30.49149 | - | - |
| R1173 | M | 45 | Clear cell renal cell carcinoma | T1a | 1 | 109.3771 | 22.64738 | - | - |
| R1145 | M | 52 | Clear cell renal cell carcinoma | T1b | 1 | 109.9364 | 25.28257 | - | - |
| R1125 | M | 55 | Clear cell renal cell carcinoma | T3a | 3 | 110.6237 | 25.95156 | - | - |
| R1121 | M | 77 | Clear cell renal cell carcinoma | T1a | 1 | 111.1772 | 27.94214 | - | - |
| R1120 | M | 53 | Clear cell renal cell carcinoma | T1b | 1 | 111.4587 | 26.92744 | - | - |
| R1201 | M | 46 | Clear cell renal cell carcinoma | T1a | 1 | 114.7102 | 26.47211 | - | - |
| R1169 | M | 56 | Clear cell renal cell carcinoma | T1b | 1 | 116.397 | 20.01842 | - | - |
| R1134 | M | 50 | Clear cell renal cell carcinoma | T1b | 1 | 116.9268 | 26.79494 | - | - |
| R1144 | F | 24 | Clear cell renal cell carcinoma | T1a | 1 | 123.425 | 17.10291 | - | - |
| R1204 | M | 32 | Clear cell renal cell carcinoma | T2a | 2 | 123.6875 | 24.79747 | - | - |
| **Healthy control-training set** | | | | | | | | | |
| **ID** | **Sex** | **Age** | **eGFR （ml/min/1.7312）** | **Urine protein (g/L)** | **BMI（kg/m2）** | **Urine leukocyte** | **Urine erythrocyte** |  |  |
| 711 | M | 60 | 82.2 | - | 23.3 | - | - |  |  |
| 1210 | M | 62 | 82.73 | - | 25.5 | - | - |  |  |
| 1409 | M | 63 | 83.08 | - | 28.1 | - | - |  |  |
| 1166 | M | 66 | 87.51 | - | 21.5 | - | - |  |  |
| 1710 | M | 69 | 88 | - | 21.5 | - | - |  |  |
| 1394 | M | 74 | 88 | - | 21.5 | - | - |  |  |
| 1853 | F | 75 | 90.38 | - | 24.8 | - | - |  |  |
| 1266 | M | 62 | 98.49 | - | 24 | - | - |  |  |
| 493 | M | 43 | 90.32 | - | 24.2 | - | - |  |  |
| 1222 | M | 54 | 90.97 | - | 24.2 | - | - |  |  |
| 1759 | M | 64 | 91 | - | 28.4 | - | - |  |  |
| 1351 | M | 52 | 91.07 | - | 22.4 | - | - |  |  |
| 567 | M | 53 | 91.07 | - | 22.4 | - | - |  |  |
| 1392 | M | 74 | 92 | - | 24.9 | - | - |  |  |
| 1458 | M | 91 | 92 | - | 24.9 | - | - |  |  |
| 1425 | F | 63 | 92.36 | - | 24.1 | - | - |  |  |
| 1716 | M | 66 | 92.57 | - | 25.5 | - | - |  |  |
| 1459 | M | 91 | 93 | - | 25.5 | - | - |  |  |
| 494 | M | 39 | 93.67 | - | 22.6 | - | - |  |  |
| 1775 | M | 61 | 94 | - | 21.4 | - | - |  |  |
| 1202 | F | 55 | 94.22 | - | 19.1 | - | - |  |  |
| 1284 | M | 64 | 95 | - | 24.7 | - | - |  |  |
| 1463 | M | 57 | 95.04 | - | 25.1 | - | - |  |  |
| 1174 | M | 57 | 95.36 | - | 22.5 | - | - |  |  |
| 424 | M | 58 | 95.36 | - | 22.5 | - | - |  |  |
| 1225 | F | 46 | 95.6 | - | 21.5 | - | - |  |  |
| 1807 | F | 74 | 95.6 | - | 21.5 | - | - |  |  |
| 278 | F | 37 | 95.99 | - | 21.7 | - | - |  |  |
| 1897 | F | 61 | 95.99 | - | 21.7 | - | - |  |  |
| 239 | M | 56 | 96.85 | - | 21.6 | - | TRACE |  |  |
| 808 | M | 57 | 97.06 | - | 21.6 | - | - |  |  |
| 326 | F | 44 | 97.15 | - | 21.7 | - | - |  |  |
| 1843 | F | 72 | 97.15 | - | 21.7 | - | - |  |  |
| 1133 | F | 65 | 97.23 | - | 24.3 | - | - |  |  |
| 226 | M | 57 | 97.39 | - | 23.1 | - | - |  |  |
| 1386 | M | 61 | 98 | - | 23.8 | - | - |  |  |
| 1756 | M | 61 | 98 | - | 24.2 | - | - |  |  |
| 1406 | F | 76 | 98.44 | - | 24.7 | - | - |  |  |
| 1147 | M | 47 | 99.1 | - | 23.7 | - | - |  |  |
| 1216 | M | 48 | 99.1 | - | 23.7 | - | - |  |  |
| 780 | M | 50 | 99.1 | - | 21.8 | - | - |  |  |
| 939 | M | 51 | 99.1 | - | 21.8 | - | - |  |  |
| 375 | M | 53 | 99.1 | - | 21.8 | - | - |  |  |
| 1109 | F | 55 | 99.11 | - | 20.9 | - | - |  |  |
| 2 | F | 46 | 99.26 | - | 22.8 | - | - |  |  |
| 1370 | F | 74 | 99.26 | - | 22.8 | - | - |  |  |
| 1218 | F | 40 | 100.07 | - | 21.3 | - | - |  |  |
| 1731 | F | 64 | 100.07 | - | 21.3 | - | - |  |  |
| 1737 | M | 60 | 101 | - | 25.3 | - | - |  |  |
| 228 | F | 39 | 101.16 | - | 22.5 | - | - |  |  |
| 1717 | F | 64 | 101.16 | - | 22.5 | - | - |  |  |
| 1040 | M | 38 | 101.64 | - | 21.2 | - | - |  |  |
| 1396 | F | 70 | 102.31 | - | 19.9 | - | - |  |  |
| 1194 | F | 57 | 102.8 | - | 23.5 | - | - |  |  |
| 385 | F | 41 | 102.93 | - | 20.6 | - | - |  |  |
| 1393 | F | 71 | 102.93 | - | 20.6 | - | - |  |  |
| 1027 | M | 54 | 103.07 | - | 23.8 | - | - |  |  |
| 1132 | M | 54 | 103.07 | - | 23.8 | - | - |  |  |
| 1114 | F | 66 | 103.21 | - | 22.9 | - | - |  |  |
| 160 | M | 20 | 103.49 | - | 23.8 | - | - |  |  |
| 1268 | F | 65 | 103.52 | - | 21.1 | - | - |  |  |
| 581 | M | 45 | 104.54 | - | 19.8 | - | - |  |  |
| 1423 | M | 52 | 104.85 | - | 20.7 | - | - |  |  |
| 470 | F | 37 | 105.38 | - | 24.2 | - | - |  |  |
| 1072 | F | 62 | 105.38 | - | 24.2 | - | - |  |  |
| 1050 | M | 55 | 105.6 | - | 22.5 | - | - |  |  |
| 1016 | M | 49 | 105.76 | - | 22.9 | - | - |  |  |
| 1230 | M | 55 | 106 | - | 26 | - | - |  |  |
| 851 | M | 52 | 107.26 | - | 23.2 | - | - |  |  |
| 803 | M | 54 | 108.22 | - | 21.5 | - | - |  |  |
| 213 | F | 58 | 108.98 | - | 23.9 | - | - |  |  |
| 789 | M | 50 | 110.4 | - | 23.4 | - | - |  |  |
| 916 | M | 39 | 110.46 | - | 21.5 | - | - |  |  |
| 1015 | M | 47 | 113.16 | - | 22.7 | - | - |  |  |
| 1869 | F | 63 | 114.42 | - | 18.5 | - | - |  |  |
| 1223 | F | 58 | 115.56 | - | 22.2 | - | - |  |  |
| 289 | M | 54 | 115.65 | - | 22.7 | - | - |  |  |
| 474 | M | 53 | 116.13 | - | 22.8 | - | - |  |  |
| 1144 | M | 53 | 116.13 | - | 22.8 | - | - |  |  |
| 198 | M | 54 | 116.62 | - | 20.5 | - | - |  |  |
| 814 | M | 44 | 117.21 | - | 22.5 | - | - |  |  |
| 285 | F | 49 | 118.02 | - | 18.8 | - | - |  |  |
| 1189 | M | 44 | 118.54 | - | 22 | - | - |  |  |
| 42 | M | 34 | 118.69 | - | 21.9 | - | - |  |  |
| 1113 | M | 45 | 119.75 | - | 21.6 | - | - |  |  |
| 1910 | F | 61 | 120.09 | - | 23.2 | - | - |  |  |
| 1186 | M | 55 | 120.24 | - | 22.5 | - | - |  |  |
| 1249 | F | 49 | 122.21 | - | 23.8 | - | - |  |  |
| 175 | M | 43 | 122.38 | - | 23.9 | - | - |  |  |
| 1232 | F | 48 | 115.21 | - | 19.1 | - | - |  |  |
| 388 | M | 43 | 126.13 | - | 22.5 | - | - |  |  |
| 264 | F | 33 | 118.32 | - | 20.7 | - | - |  |  |
| 149 | F | 47 | 118.32 | - | 20.7 | - | - |  |  |
| 179 | M | 23 | 120.03 | - | 21.2 | - | - |  |  |
| 102 | M | 35 | 120.03 | - | 21.2 | - | - |  |  |
| 50 | M | 48 | 123.52 | - | 22.8 | - | - |  |  |
| 1839 | M | 72 | 90.65 | - | 28.4 | - | - |  |  |
| 1930 | M | 59 | 90.97 | - | 24.2 | - | - |  |  |
| 1710 | M | 69 | 92 | - | 21.4 | - | - |  |  |
| 1926 | M | 68 | 94.31 | - | 21.4 | - | - |  |  |
| 1716 | M | 66 | 95.04 | - | 25.1 | - | - |  |  |
| 1908 | M | 69 | 95.15 | - | 24.7 | - | - |  |  |
| 1904 | M | 64 | 95.36 | - | 22.5 | - | - |  |  |
| 1917 | M | 61 | 96.85 | - | 21.6 | - | - |  |  |
| 1859 | M | 68 | 97 | - | 22.9 | - | - |  |  |
| 1759 | M | 64 | 97.06 | - | 21.6 | - | - |  |  |
| 1815 | M | 62 | 97.39 | - | 23.1 | - | - |  |  |
| 1800 | M | 63 | 98 | - | 29.8 | - | - |  |  |
| 55 | M | 33 | 98.56 | - | 21.4 | - | - |  |  |
| 1925 | M | 51 | 99.1 | - | 21.8 | - | - |  |  |
| 1913 | M | 60 | 102 | - | 25.1 | - | - |  |  |
| 1882 | M | 61 | 103 | - | 27.8 | - | - |  |  |
| 1868 | M | 52 | 104.85 | - | 20.7 | - | - |  |  |
| 1931 | M | 59 | 105.6 | - | 22.5 | - | - |  |  |
| 50 | M | 48 | 105.76 | - | 22.9 | - | - |  |  |
| 1854 | M | 54 | 107.26 | - | 23.2 | - | - |  |  |
| 1879 | M | 57 | 108.22 | - | 21.5 | - | - |  |  |
| 462 | F | 24 | 108.54 | - | 21.3 | - | - |  |  |
| 1933 | M | 51 | 110.4 | - | 23.4 | - | - |  |  |
| 1934 | M | 54 | 115.65 | - | 22.7 | - | - |  |  |
| 1819 | M | 54 | 116.13 | - | 22.8 | - | - |  |  |
| 1909 | M | 54 | 116.62 | - | 20.5 | - | - |  |  |
| 503 | M | 25 | 119.13 | - | 20.9 | - | - |  |  |
| 998 | F | 24 | 119.51 | - | 23.8 | - | - |  |  |
| 1887 | M | 60 | 120.24 | - | 22.5 | - | - |  |  |
| 155 | F | 27 | 121.52 | - | 20.4 | - | - |  |  |
| 136 | F | 20 | 125.54 | - | 18.4 | - | - |  |  |
| 56 | F | 27 | 117.92 | - | 23.7 | - | - |  |  |
| 486 | F | 20 | 118.13 | - | 19.1 | - | - |  |  |
| **ID** | **Sex** | **Age** | **Pathology** | **eGFR （ml/min/1.7312）** | **Urine leukocyte** | **Urine erythrocyte** | **BMI** |  |  |
| R904 | M | 63 | CY | 83.55458 | - | - | 27.30997 |  |  |
| R983 | F | 50 | CY | 89.23472 | - | - | 20.78826 |  |  |
| R938 | M | 50 | CY | 94.14312 | - | - | 25.35154 |  |  |
| R1092 | F | 64 | CY | 81.02054 | - | - | 28.84153 |  |  |
| R1111 | M | 47 | CY | 81.95708 | - | - | 24.33748 |  |  |
| R1035 | F | 54 | CY | 88.52519 | - | - | 32.34682 |  |  |
| R1174 | F | 58 | AML | 88.72887 | - | - | 21.48438 |  |  |
| R1166 | F | 58 | AML | 90.10703 | - | - | 22.94213 |  |  |
| R960 | M | 66 | CY | 90.40081 | - | - | 22.85714 |  |  |
| R912 | F | 43 | AML | 90.86755 | - | TRACE | 23.82813 |  |  |
| R902 | F | 40 | AML | 93.45777 | - | - | 22.03857 |  |  |
| R1123 | F | 42 | AML | 94.06018 | - | - | 19.0274 |  |  |
| R1193 | M | 43 | AML | 94.18576 | - | - | 26.65262 |  |  |
| R1195 | F | 41 | AML | 85.95109 | - | - | 21.71925 |  |  |
| R958 | F | 32 | AML | 88.83098 | - | - | 19.26717 |  |  |
| R1026 | M | 57 | CY | 88.98651 | - | - | 23.1405 |  |  |
| R876 | M | 55 | CY | 89.63408 | - | - | 27.68166 |  |  |
| R877 | F | 53 | AML | 94.70575 | - | - | 25.03992 |  |  |
| R1013 | F | 47 | AML | 97.04395 | - | - | 20.22913 |  |  |
| R1179 | M | 40 | CY | 97.05446 | - | - | 32.40741 |  |  |
| R1053 | M | 53 | CY | 97.4858 | - | - | 26.23457 |  |  |
| R1091 | F | 54 | AML | 98.2461 | - | - | 25.39063 |  |  |
| R1039 | F | 27 | AML | 98.71756 | - | - | 24.02381 |  |  |
| R1009 | M | 34 | CY | 98.82722 | - | - | 29.62963 |  |  |
| R1187 | F | 28 | AML | 99.8176 | - | - | 21.29529 |  |  |
| R1031 | M | 44 | CY | 101.2391 | - | - | 28.01022 |  |  |
| R991 | M | 42 | CY | 107.2707 | - | - | 23.70242 |  |  |
| R883 | F | 44 | CY | 109.1352 | - | - | 21.875 |  |  |
| R899 | F | 35 | CY | 109.5408 | - | - | 24.22145 |  |  |
| R1032 | F | 61 | AML | 120.2696 | - | - | 28.44095 |  |  |
| R929 | M | 60 | CY | 121.5391 | - | - | 32.11195 |  |  |
| R1036 | F | 26 | AML | 124.1346 | - | - | 26.98962 |  |  |
| R910 | M | 53 | CY | 124.6387 | - | - | 23.2072 |  |  |
| R1028 | F | 15 | AML | 122.2304 | - | - | 21.28906 |  |  |
| R1122 | F | 42 | AML | 98.24 | - | - | 25.13673 | Cohort 2-validation | |
| R11662 | F | 58 | AML | 90.10703 | - | - | 22.94213 | Cohort 2-validation | |
| R1178 | M | 61 | CY | 101.2391 | - | - | 21.48438 | Cohort 2-validation | |
| R1179 | M | 40 | CY | 97.05446 | - | - | 26.40741 | Cohort 2-validation | |
| RV11791 | M | 40 | CY | 97.05446 | - | - | 26.40741 | Cohort 2-validation | |
| RV11782 | M | 50 | AML | 90.25 | - | - | 23.70242 | Cohort 2-validation | |
| R9101 | M | 53 | CY | 124.6387 | - | - | 25.35388 | Cohort 2-validation | |

Note: “-”, negative.

Table S2 Metabolic pathways shifted in RCC compared with controls

| Name | Metabolites total | Hits.total | Hits.sig | P-value |
| --- | --- | --- | --- | --- |
| Galactose metabolism | 41 | 24 | 24 | 9.85E-04 |
| Linoleate metabolism | 46 | 16 | 16 | 1.01E-02 |
| Leukotriene metabolism | 92 | 42 | 37 | 3.03E-02 |
| Tryptophan metabolism | 94 | 58 | 49 | 5.89E-02 |
| Hexose phosphorylation | 20 | 12 | 12 | 3.21E-02 |
| Starch and Sucrose Metabolism | 33 | 12 | 12 | 3.21E-02 |
| Caffeine metabolism | 11 | 11 | 11 | 4.28E-02 |
| Ascorbate (Vitamin C) and Aldarate Metabolism | 29 | 11 | 11 | 4.28E-02 |

*Note:* This table corresponds to Fig2C. Metabolites total: the number of all metabolites involved in the pathway; Hits.total: the number of predicted metabolites from our data in this pathway; Hits.sig: the number of predicted metabolites showing significant changes from our data in this pathway. P-value: pathway enrichment confidence.Table S3 Differential metabolites between RCC and control

| Variables | Metabolites ID | Description | Score | FC (RCC/control) | VIP |
| --- | --- | --- | --- | --- | --- |
| 3.26_216.0080n | HMDB04148 | Dopamine 4-sulfate | 46.9 | 1.95 | 1.00 |
| 3.26_237.0855m/z | HMDB01200 | N'-Formylkynurenine | 45.7 | 1.67 | 2.81 |
| 3.62_265.0838m/z | HMDB28759 | Aspartyl-Methionine | 45.6 | 1.58 | 1.48 |
| 4.24_265.1260m/z | HMDB28829 | Glutamyl-Threonine | 45.3 | 1.82 | 1.40 |
| 4.39_167.0554m/z | HMDB01886 | 3-Methylxanthine | 43.3 | 0.64 | 1.51 |
| 4.53_254.1485m/z | HMDB62497 | N-acetyl-5-methoxykynuramine | 46.5 | 0.59 | 1.14 |
| 4.69_289.1742m/z | HMDB33917 | 4-Hydroxy-3-methoxy-2,10-bisaboladien-9-one | 38.7 | 2.91 | 1.98 |
| 4.83_213.0857m/z | HMDB38055 | 2-Hydroxy-7-methoxy-2H-1,4-benzoxazin-3(4H)-one | 43.3 | 0.56 | 1.30 |
| 4.94_253.0916m/z | HMDB29956 | Nebularine | 41.2 | 0.34 | 1.09 |
| 5.22_207.1248n | HMDB37700 | Propyl cinnamate | 42.5 | 0.47 | 1.40 |
| 5.35_216.1217m/z | HMDB30991 | 5-(3E-Pentenyl)tetrahydro-2-oxo-3-furancarboxylic acid | 42.8 | 1.59 | 1.09 |
| 5.35_254.1134m/z | HMDB29122 | Valyl-Asparagine | 36.3 | 0.56 | 1.54 |
| 5.38_383.2155m/z | HMDB06468 | 4-Hydroxydebrisoquine | 41.9 | 2.33 | 1.71 |
| 5.58_259.2001m/z | HMDB38588 | Dioscoretine | 45.1 | 1.75 | 1.06 |
| 5.59_396.1753m/z | HMDB39503 | N-Jasmonoyltyrosine | 44.6 | 2.51 | 1.39 |
| 5.69_228.0853m/z | HMDB29162 | Gamma-glutamyl-Valine | 48.3 | 1.59 | 1.31 |
| 5.72_425.2259m/z | HMDB35844 | Lucidone A | 46.3 | 2.27 | 1.29 |
| 5.73_409.2310m/z | HMDB39948 | 3,4-Dihydroxy-2-hydroxymethyl-1-pyrrolidinepropanamide | 41.9 | 2.66 | 1.52 |
| 5.73_411.2466m/z | HMDB62744 | 7alpha-hydroxy-3-oxochol-4-en-24-oic Acid | 45.7 | 2.98 | 1.78 |
| 5.80_210.1112m/z | HMDB30580 | (R)-Shinanolone | 47.3 | 2.29 | 1.30 |
| 5.85_314.1944m/z | HMDB38120 | Ovalicin | 46.5 | 5.48 | 2.00 |
| 5.85_363.2238n | HMDB61062 | 7-hydroxygranisetron | 45.1 | 1.79 | 1.37 |
| 5.87_300.1788m/z | HMDB38989 | Oxyhumulinic acid | 44.8 | 2.21 | 1.24 |
| 5.90_342.1891m/z | HMDB14716 | Valaciclovir | 45.7 | 1.65 | 1.14 |
| 5.91_381.1737m/z | HMDB15613 | Ecabet | 44.7 | 0.44 | 1.40 |
| 6.08_262.0940n | HMDB28760 | Aspartyl-Phenylalanine | 40.2 | 1.87 | 1.70 |
| 6.31_292.1560m/z | HMDB39660 | Gossyvertin | 43.4 | 0.56 | 1.25 |
| 6.32_225.1102m/z | HMDB59754 | 3-Methylazelaic acid | 46.3 | 1.98 | 1.30 |
| 6.38_269.1350m/z | HMDB00053 | Androstenedione | 42.2 | 1.76 | 1.07 |
| 6.45_107.0848m/z | HMDB59851 | o-Xylene | 41.1 | 1.76 | 1.31 |
| 6.68_179.0805m/z | HMDB37688 | 5-Nitro-2-propoxyaniline | 46.6 | 0.54 | 1.28 |
| 6.88_239.1376m/z | HMDB35965 | 5beta-1,3,7(11)-Eudesmatrien-8-one | 43.1 | 1.75 | 1.16 |
| 6.90_195.1367m/z | HMDB33601 | Dihydrojasmonic acid | 43.6 | 2.15 | 1.28 |
| 6.92_177.1264m/z | HMDB32567 | Hexylresorcinol | 42.5 | 5.60 | 1.25 |
| 6.92_230.1505n | HMDB30143 | Talaromycin A | 39.9 | 2.60 | 1.19 |
| 6.92_424.2153m/z | HMDB15637 | Vilazodone | 43.1 | 3.01 | 1.60 |
| 7.00_369.1498m/z | HMDB00796 | N-Acetyl-4-O-acetylneuraminic acid | 44.9 | 0.54 | 1.30 |
| 7.02_395.2516m/z | HMDB13627 | Cervonoyl ethanolamide | 42.2 | 1.81 | 1.34 |
| 7.17_221.1522m/z | HMDB32248 | 11-Dodecenoic acid | 44.7 | 1.95 | 1.11 |
| 7.21_330.1658n | HMDB32959 | 1-Octen-3-yl glucoside | 43.7 | 0.64 | 1.75 |
| 7.25_525.2392n | HMDB10320 | Cortolone-3-glucuronide | 42.4 | 0.63 | 1.10 |
| 7.28_432.1968n | HMDB60897 | Diphenhydramine N-glucuronide | 41.9 | 2.76 | 1.40 |
| 7.35_386.2513m/z | HMDB05099 | Thromboxane B3 | 40.6 | 0.32 | 1.85 |
| 7.36_240.1710n | HMDB35593 | Rishitin | 47.6 | 4.11 | 1.76 |
| 7.36_434.2124n | HMDB61126 | 4,5-Dihydro-drospirenone-3-sulfate | 42.8 | 4.62 | 1.97 |
| 7.44_540.2535n | HMDB10357 | Tetrahydroaldosterone-3-glucuronide | 44.3 | 0.55 | 1.11 |
| 7.69_349.2351m/z | HMDB40954 | 9alpha-(3-Methyl-2E-pentenoyloxy)-4S-hydroxy-10(14)-oplopen-3-one | 44.5 | 0.49 | 1.30 |
| 7.72_454.2619m/z | HMDB14984 | Flurandrenolide | 49.2 | 2.65 | 2.16 |
| 7.76_302.2310m/z | HMDB06320 | 2,6 Dimethylheptanoyl carnitine | 51.4 | 0.60 | 1.56 |
| 7.88_524.3032m/z | HMDB38327 | (3b,9R)-5-Megastigmene-3,9-diol 9-[apiosyl-(1->6)-glucoside] | 46.4 | 2.33 | 1.09 |
| 7.98_342.1711m/z | HMDB32671 | (E)-2',4,4'-Trihydroxy-3-prenylchalcone | 42 | 0.48 | 1.70 |
| 8.00_478.2615m/z | HMDB37530 | 1'-O-Acetylpaxilline | 42 | 1.93 | 1.29 |
| 8.18_296.1839m/z | HMDB01518 | Alpha-CEHC | 41 | 3.84 | 1.59 |
| 8.27_462.2436n | HMDB29349 | Neryl rhamnosyl-glucoside | 42.9 | 1.58 | 1.28 |
| 8.38_258.1637n | HMDB39152 | Flavidulol A | 39.6 | 0.49 | 1.03 |
| 8.40_542.2859m/z | HMDB33617 | Vignatic acid B | 36.1 | 0.36 | 1.05 |
| 8.43_277.2146m/z | HMDB04590 | 19-Nor-5-androstenediol | 45.3 | 2.36 | 1.69 |
| 8.43_372.2182m/z | HMDB31877 | Acetylsalvipisone | 44.6 | 0.49 | 1.47 |
| 8.43_488.2591n | HMDB00722 | Lithocholyltaurine | 44 | 3.58 | 2.24 |
| 8.52_374.2339m/z | HMDB35099 | gamma-Crocetin | 44.3 | 0.48 | 2.34 |
| 8.59_490.2749n | HMDB60119 | 12-O-D-Glucuronoside-13-hydroxyoctadec-9Z-enoate | 45.9 | 3.20 | 1.95 |
| 8.71_368.2774m/z | HMDB41997 | Pregnanetriolone | 45.4 | 0.30 | 1.00 |
| 8.74_490.2750n | HMDB60118 | 12-Hydroxy-13-O-D-glucuronoside-octadec-9Z-enoate | 46.5 | 2.25 | 1.87 |
| 8.76_386.2879m/z | HMDB37961 | gamma-Eudesmol rhamnoside | 45.8 | 0.36 | 1.85 |
| 9.00_384.2126n | HMDB12643 | 20-Trihydroxy-leukotriene-B4 | 42.4 | 1.76 | 1.16 |

*Note:* Score : the confidence of metabolite annotation by Progenesis QI. The highest score is 60, and the cutoff is 35. FC, fold change. VIP, Variable importance for the projection. VIP represents the contribution weight of variable for group separation. The cutoff is 1.Table S4 AUC values of metabolites for distinction of RCC and Control.

| Name | AUC |
| --- | --- |
| gamma-Eudesmol rhamnoside | 0.85 |
| N-Jasmonoyltyrosine | 0.85 |
| Vignatic acid B | 0.84 |
| E-2',4,4'-Trihydroxy-3-prenylchalcone | 0.84 |
| Thromboxane B3 | 0.82 |
| Aspartyl-Phenylalanine | 0.82 |
| Nebularine | 0.82 |
| Tetrahydroaldosterone-3-glucuronide | 0.81 |
| 4,5-Dihydro-drospirenone-3-sulfate | 0.81 |
| 2,6 Dimethylheptanoyl carnitine | 0.80 |
| Androstenedione | 0.80 |
| Flavidulol A | 0.80 |
| Dopamine 4-sulfate | 0.79 |
| Pregnanetriolone | 0.79 |
| 3-Methylazelaic acid | 0.79 |
| Cortolone-3-glucuronide | 0.79 |
| Rishitin | 0.79 |
| Talaromycin A | 0.78 |
| Diphenhydramine N-glucuronide | 0.78 |
| 7alpha-hydroxy-3-oxochol-4-en-24-oic Acid | 0.78 |
| 9alpha-3-Methyl-2E-pentenoyloxy-4S-hydroxy-1014-oplopen-3-one | 0.78 |
| Vilazodone | 0.77 |
| Lithocholyltaurine | 0.77 |
| 7-hydroxygranisetron | 0.77 |
| 11-Dodecenoic acid | 0.76 |
| 3,4-Dihydroxy-2-hydroxymethyl-1-pyrrolidinepropanamide | 0.76 |
| o-Xylene | 0.75 |
| N-acetyl-5-methoxykynuramine | 0.75 |
| 20-Trihydroxy-leukotriene-B4 | 0.75 |
| Dihydrojasmonic acid | 0.75 |
| Flurandrenolide | 0.75 |
| Oxyhumulinic acid | 0.74 |
| Ecabet | 0.74 |
| 1-Octen-3-yl glucoside | 0.74 |
| 12-O-D-Glucuronoside-13-hydroxyoctadec-9Z-enoate | 0.74 |
| Ovalicin | 0.73 |
| Acetylsalvipisone | 0.73 |
| Lucidone A | 0.73 |
| Alpha-CEHC | 0.72 |
| Gamma-glutamyl-Valine | 0.71 |
| 19-Nor-5-androstenediol | 0.71 |
| 4-Hydroxydebrisoquine | 0.70 |
| 12-Hydroxy-13-O-D-glucuronoside-octadec-9Z-enoate | 0.70 |
| Hexylresorcinol | 0.70 |
| Gossyvertin | 0.69 |
| gamma-Crocetin | 0.69 |
| N'-Formylkynurenine | 0.69 |
| Valyl-Asparagine | 0.69 |
| N-Acetyl-4-O-acetylneuraminic acid | 0.68 |
| 4-Hydroxy-3-methoxy-2,10-bisaboladien-9-one | 0.68 |
| R-Shinanolone | 0.68 |
| 1'-O-Acetylpaxilline | 0.67 |
| Propyl cinnamate | 0.67 |
| Cervonoyl ethanolamide | 0.67 |
| 5beta-1,3,711-Eudesmatrien-8-one | 0.67 |
| 2-Hydroxy-7-methoxy-2H-1,4-benzoxazin-34H-one | 0.66 |
| Dioscoretine | 0.66 |
| Neryl rhamnosyl-glucoside | 0.65 |
| 5-3E-Pentenyltetrahydro-2-oxo-3-furancarboxylic acid | 0.65 |
| 3-Methylxanthine | 0.64 |
| Glutamyl-Threonine | 0.64 |
| Valaciclovir | 0.64 |
| 5-Nitro-2-propoxyaniline | 0.63 |
| 3b,9R-5-Megastigmene-3,9-diol 9-apiosyl-1-6-glucoside | 0.62 |
| Aspartyl-Methionine | 0.62 |

Table S5. Metabolites panel for RCC and control distinction

| Name | AUC | *p*-value |
| --- | --- | --- |
| N-Jasmonoyltyrosine | 0.85 | 1.06E-11 |
| Tetrahydroaldosterone-3-glucuronide | 0.81 | 3.57E-11 |
| Androstenedione | 0.80 | 1.21E-06 |
| Dopamine 4-sulfate | 0.79 | 4.90E-07 |
| 3-Methylazelaic acid | 0.79 | 7.10E-05 |
| Cortolone-3-glucuronide | 0.79 | 7.34E-08 |
| 7alpha-hydroxy-3-oxochol-4-en-24-oic Acid | 0.78 | 2.76E-08 |
| Cortolone-3-glucuronide | 0.78 | 2.45E-08 |
| Lithocholyltaurine | 0.77 | 8.68E-08 |
| 11-Dodecenoic acid | 0.76 | 2.25E-08 |
| Combined-testing | 0.905 |  |
| Combined-validation | 0.885 |  |

TableS6 Metabolic pathways shifted in RCC compared with benign

| **Name** | **Metabolites total** | **Hits.total** | **Hits.sig** | **P-value** |
| --- | --- | --- | --- | --- |
| Vitamin B9 (folate) metabolism | 41 | 24 | 24 | 0.007384 |
| Tryptophan metabolism | 46 | 16 | 16 | 0.037589 |
| Biopterin metabolism | 92 | 42 | 37 | 0.03784 |
| Pyrimidine metabolism | 94 | 58 | 49 | 0.12583 |
| Urea cycle/amino group metabolism | 20 | 12 | 12 | 0.16284 |

*Note:* This table corresponds to Fig3B. Metabolites total: the number of all metabolites involved in the pathway; Hits.total: the number of predicted metabolites from our data in this pathway; Hits.sig: the number of predicted metabolites showing significant changes from our data in this pathway. P-value: pathway enrichment confidence.

TableS7 Differential metabolites between RCC and benign

| **Variables** | **Metabolites ID** | **Description** | **Score** | **Log2 FC** | **VIP** |
| --- | --- | --- | --- | --- | --- |
| 2.37_225.0857m/z | HMDB11631 | L-3-Hydroxykynurenine | 44.3 | -0.39126 | 2.0744 |
| 3.26_237.0855m/z | HMDB01200 | N'-Formylkynurenine | 44.4 | -0.89228 | 3.11515 |
| 3.43_386.1111m/z | HMDB38960 | Zeanoside C | 38.1 | 0.4381 | 2.33184 |
| 3.54_220.0837n | HMDB00472 | 5-Hydroxy-L-tryptophan | 51.7 | -0.65997 | 1.16039 |
| 4.60_191.0572n | HMDB60400 | 5-Phenyl-1,3-oxazinane-2,4-dione | 48.8 | 1.6909 | 2.30773 |
| 4.69_241.0918m/z | HMDB62179 | AICA-riboside | 52.2 | -0.41008 | 1.99709 |
| 4.71_366.1409n | HMDB33108 | Semilepidinoside B | 50.4 | -1.0188 | 1.26122 |
| 5.12_311.1213n | HMDB01961 | 1,7-Dimethylguanosine | 41.6 | -1.5144 | 1.10177 |
| 5.13_198.0749m/z | HMDB38336 | 2-Hydroxy-3-(3,4-dihydroxyphenyl)propanamide | 40.5 | -0.2657 | 1.88693 |
| 5.30_362.1536m/z | HMDB15296 | Ofloxacin | 39 | -0.48188 | 1.30956 |
| 5.69_396.1379m/z | HMDB41231 | Gibberellin A99 | 40.2 | -0.78245 | 1.37034 |
| 5.72_338.1328m/z | HMDB30181 | Erythratine | 41.6 | -0.32685 | 1.5412 |
| 6.25_400.1711n | HMDB36460 | Safynol | 34.1 | 0.60739 | 3.56235 |
| 6.28_184.1090n | HMDB37011 | 5-Hydroxy-p-mentha-6,8-dien-2-one | 44.3 | -1.0127 | 2.16007 |
| 6.46_277.1167m/z | HMDB00594 | Glutamylphenylalanine | 37.6 | -0.42471 | 1.33561 |
| 6.57_316.1273m/z | HMDB29009 | Phenylalanyl-Gamma-glutamate | 41.3 | -1.2936 | 1.31923 |
| 7.03_541.2607m/z | HMDB10357 | Tetrahydroaldosterone-3-glucuronide | 42.1 | 0.64873 | 2.44319 |
| 7.18_235.1314m/z | HMDB36047 | 3alpha-Hydroxyoreadone | 48.1 | -0.29033 | 1.59907 |
| 7.26_268.1294n | HMDB61643 | 3-carboxy-4-methyl-5-pentyl-2-furanpropanoic acid | 40.4 | 0.49521 | 1.87604 |
| 7.68_625.3423n | HMDB02579 | Glycochenodeoxycholic acid 3-glucuronide | 43.2 | 0.648 | 2.13792 |
| 8.11_252.1346n | HMDB30143 | Talaromycin A | 48.2 | -1.6434 | 1.82984 |
| 8.13_195.1366m/z | HMDB33601 | Dihydrojasmonic acid | 42 | -0.37556 | 2.164 |

*Note:* Score : the confidence of metabolite annotation by Progenesis QI. The highest score is 60, and the cutoff is 35. FC, fold change. VIP, Variable importance for the projection. VIP represents the contribution weight of variable for group separation. The cutoff is 1.

TableS8 AUC values of metabolites for distinction of RCC and benign

| **Name** | **AUC** |
| --- | --- |
| Erythratine | 0.79616 |
| 5-Phenyl-1,3-oxazinane-2,4-dione | 0.77655 |
| AICA-riboside | 0.77612 |
| Safynol | 0.77612 |
| Gibberellin A99 | 0.74072 |
| L-3-Hydroxykynurenine | 0.73817 |
| 1,7-Dimethylguanosine | 0.73774 |
| Semilepidinoside B | 0.73561 |
| Dihydrojasmonic acid | 0.73177 |
| 5-Hydroxy-p-mentha-6,8-dien-2-one | 0.72623 |
| Ofloxacin | 0.7258 |
| Tetrahydroaldosterone-3-glucuronide | 0.72495 |
| 5-Hydroxy-L-tryptophan | 0.72196 |
| N'-Formylkynurenine | 0.72154 |
| Phenylalanyl-Gamma-glutamate | 0.72154 |
| Talaromycin A | 0.71557 |
| Glycochenodeoxycholic acid 3-glucuronide | 0.71322 |
| Zeanoside C | 0.71173 |
| Glutamylphenylalanine | 0.71045 |
| 3alpha-Hydroxyoreadone | 0.70917 |
| 2-Hydroxy-3-3,4-dihydroxyphenylpropanamide | 0.70704 |
| 3-carboxy-4-methyl-5-pentyl-2-furanpropanoic acid | 0.70618 |

Table S9 Differential metabolites between early- and late-RCC

| Variables | Compound ID | Description | Score | FC(Late/early) | VIP |
| --- | --- | --- | --- | --- | --- |
| 2.37_225.0857m/z | HMDB00273 | Thymidine | 43.8 | 4.28 | 1.13 |
| 2.81_204.1332m/z | HMDB28695 | Alanyl-Proline | 43.4 | 1.86 | 1.54 |
| 4.85_329.1689m/z | HMDB33939 | Domoic acid | 43 | 9.40 | 1.43 |
| 4.94_294.1066m/z | HMDB14642 | Flutamide | 41.8 | 2.76 | 1.33 |
| 4.95_199.1066m/z | HMDB29069 | Threoninyl-Proline | 47.5 | 1.83 | 1.73 |
| 5.38_385.1772m/z | HMDB60534 | Desmethylazelastine | 38.8 | 8.49 | 1.43 |
| 5.40_420.1478m/z | HMDB59973 | 4-Hydroxy-5-(3',5'-dihydroxyphenyl)-valeric acid-O-glucuronide | 41.9 | 4.73 | 1.31 |
| 5.59_231.1325m/z | HMDB29128 | Valyl-Hydroxyproline | 41.4 | 0.64 | 1.17 |
| 5.60_227.1376m/z | HMDB28908 | Isoleucyl-Hydroxyproline | 43.5 | 0.59 | 1.21 |
| 5.72_425.2259m/z | HMDB35844 | Lucidone A | 40.8 | 1.86 | 1.56 |
| 5.73_409.2310m/z | HMDB29052 | Serinyl-Valine | 40.9 | 1.91 | 1.41 |
| 5.81_267.1454n | HMDB32116 | Helinorbisabone | 43.5 | 25.15 | 1.91 |
| 6.52_490.1444n | HMDB40512 | 4',5,6-Trimethylscutellarein 7-glucoside | 42.9 | 3.28 | 1.44 |
| 6.59_479.2391m/z | HMDB00907 | Sulfolithocholic acid | 38.1 | 4.32 | 1.47 |
| 6.67_219.1002m/z | HMDB40358 | (R)-Bitalin A | 46.7 | 2.51 | 1.32 |
| 6.68_399.1639m/z | HMDB00768 | N-Acetyl-9-O-lactoylneuraminic acid | 44 | 21.26 | 1.48 |
| 6.74_246.1242n | HMDB34719 | Isoamberboin | 44.6 | 1.95 | 1.29 |
| 6.79_460.2096m/z | HMDB34120 | Lusitanicoside | 48.4 | 3.09 | 1.44 |
| 7.03_541.2607m/z | HMDB10357 | Tetrahydroaldosterone-3-glucuronide | 42.5 | 1.65 | 1.40 |
| 7.88_355.2610m/z | HMDB13627 | Cervonoyl ethanolamide | 41.8 | 4.07 | 1.14 |
| 7.88_584.3161n | HMDB02577 | Cholic acid glucuronide | 51.5 | 4.27 | 1.14 |
| 8.05_237.1834m/z | HMDB29604 | Lubiminol | 47.5 | 0.58 | 1.19 |
| 8.24_477.2818m/z | HMDB34617 | (3b,4b,11b,14b)-11-Ethoxy-3,4-epoxy-14-hydroxy-12-cyathen-15-al 14-xyloside | 40.5 | 1.51 | 1.43 |
| 8.36_459.2494m/z | HMDB36162 | 3'-Hydroxy-HT2 toxin | 53.2 | 2.43 | 1.76 |
| 8.58_251.1990m/z | HMDB00806 | Myristic acid | 40.6 | 2.24 | 1.46 |

*Note:* Score : the confidence of metabolite annotation by Progenesis QI. The highest score is 60, and the cutoff is 35. FC, fold change. VIP, Variable importance for the projection. VIP represents the contribution weight of variable for group separation. The cutoff is 1.

Table S10 AUC values of metabolites for distinction of early- and late-RCC

| Name | AUC |
| --- | --- |
| Domoic acid | 0.84 |
| Cervonoyl ethanolamide | 0.75 |
| R-Bitalin A | 0.75 |
| Thymidine | 0.74 |
| 3b,4b,11b,14b-11-Ethoxy-3,4-epoxy-14-hydroxy-12-cyathen-15-al 14-xyloside | 0.72 |
| Cholic acid glucuronide | 0.72 |
| Alanyl-Proline | 0.72 |
| Lubiminol | 0.72 |
| Lucidone A | 0.72 |
| Isoleucyl-Hydroxyproline | 0.71 |
| Valyl-Hydroxyproline | 0.71 |
| Flutamide | 0.70 |
| Serinyl-Valine | 0.70 |
| Tetrahydroaldosterone-3-glucuronide | 0.69 |
| Myristic acid | 0.69 |
| 4',5,6-Trimethylscutellarein 7-glucoside | 0.68 |
| 4-Hydroxy-5-3',5'-dihydroxyphenyl-valeric acid-O-glucuronide | 0.66 |
| Threoninyl-Proline | 0.66 |
| 3'-Hydroxy-HT2 toxin | 0.65 |
| Isoamberboin | 0.65 |
| Sulfolithocholic acid | 0.64 |
| Desmethylazelastine | 0.62 |
| Lusitanicoside | 0.61 |
| N-Acetyl-9-O-lactoylneuraminic acid | 0.58 |
| Helinorbisabone | 0.58 |
